# Awareness and perspectives of translational assessment tools in preclinical research: insights from a survey

**DOI:** 10.1016/j.namjnl.2026.100146

**Published:** 2026-10-13

**Authors:** Francesca Pistollato, Ignacio J. Tripodi, Fabia Furtmann, Marco Straccia, Helder Constantino

**Affiliations:** a Humane World for Animals International, Belgium; b Independent Scholar, Barcelona, Spain; c FRESCI by SCIENCE&STRATEGY SL, Vacarisses, Spain

**Keywords:** Translational relevance, Tools, Awareness, Preclinical research, Animal models, Non-animal methods, Replacement

## Abstract

Insufficient translation of preclinical findings into clinical benefit continues to undermine drug development, contributing to high attrition rates and avoidable ethical and economic costs. In response, a range of tools and frameworks has emerged to support structured evaluation of translational relevance, including assessment of animal model performance, study validity, bias and harm-benefit considerations. How widely these approaches are recognised and applied in practice remains poorly understood. To address this gap, the Biomedical Research for the 21st Century (BioMed21) Collaboration conducted an exploratory online survey targeting key preclinical research stakeholders across different countries and professional backgrounds. The survey collected responses from 105 self-selected participants across academia, industry, governmental and public institutions, and other organisations, focusing on awareness of existing tools, perceived value and obstacles to their implementation.

While respondents overwhelmingly acknowledged the importance of structured assessment approaches, familiarity with the available tools and frameworks was limited, with more than one-third-reporting no prior exposure. Perceived barriers to broader adoption included insufficient standardisation and reproducibility, limited availability of robust comparative human data, uncertainty regarding the validity of assessment outcomes and reliance on subjective expert opinion. Despite these challenges, participants recognised the potential of such tools to support more informed model selection, improve preclinical decision-making and increase research robustness. Relevance to human biology emerged as a particularly important criterion for evaluating translational value.

Building on these findings, we discuss potential future strategies to improve accessibility and application of translational assessment frameworks, and support more human-relevant biomedical research.

## Highlights

• Awareness of existing translational assessment tools remains limited.
• Respondents recognised the value of structured translational assessment.
• Time, expertise and complexity were perceived barriers to wider adoption.
• Model relevance should be assessed in relation to the research question.
• Greater accessibility and integration of assessment tools are needed.

## 1 Introduction

Drug discovery and development remain notoriously failure-prone, with approximately 90% of drug candidates that enter clinical testing ultimately failing to reach the market (Sun et al., 2022). Although this attrition is multifactorial, a major contributing factor is the limited translatability of preclinical findings to human outcomes. Economically, the high cost of failed drug development, which ranges from several hundred millions to billions USD per approved drug depending on the therapeutic area and modeling assumptions (Sertkaya et al., 2024), places a substantial burden on healthcare systems. Beyond financial considerations, the foremost ethical concern relates to opportunity costs for patients, many of whom continue to lack access to effective treatments, as well as avoidable animal suffering.

Several factors undermine the translatability of preclinical research, including the limited capacity of non-human animal models and overly simplistic in vitro systems to capture the complexity of human physiology, disease mechanisms, systemic interactions, and population-level heterogeneity. This biological mismatch has contributed to the progression of drug candidates with limited efficacy or unanticipated toxicity in humans (Hartung, 2024).

Compounding these challenges, approval rates for animal research proposals are extremely high (Azilagbetor et al., 2024; Busquet et al., 2020), suggesting that current ethical and scientific review processes may not consistently apply sufficiently rigorous scrutiny. Such permissive review environments may consequently affect the overall quality and reliability of preclinical evidence, with downstream consequences for research translatability (Ingber, 2022; Tagle, 2025).

In an effort to improve translatability of preclinical research and enhance the efficiency of drug discovery, advanced human-relevant methods, including microphysiological systems, organoids, and in silico technologies, which are collectively referred as to new approach methods or non-animal methods (NAMs), have been developed and are applied in both basic and applied research (El-Tanani et al., 2025; Mirlohi et al., 2025). A transition towards NAM-based research has also received policy-level support, particularly from the European Commission (EC), with its European Research Area (ERA) action aimed at supporting human-relevant NAM-based research (European Commission, 2025). This is in line with global regulatory movements in support of Replacement, Reduction and Refinement of animal studies (3Rs), fostered by the European Medicines Agency (EMA) (European Medicines Agency, 2023, 2016), the US Food and Drug Administration (FDA) (Booker, 2025; Rand, 2022; U.S. Food and Drug Administration (FDA), 2025a, 2025b), and other regulatory bodies worldwide (Parvatam, 2023).

In parallel, to enable a more objective and systematic assessment of preclinical research translatability, grading of evidence, the identification of potential sources of bias, and operationalise harm-benefit analysis (HBA), several tools and evaluation frameworks have been developed over the last 15 years. Such tools include, for example, the Clinical relevance assessment of animal preclinical research (RAA) tool (Gurusamy et al., 2021), the Preclinical assessment for translation to humans (PATH) approach (Kimmelman et al., 2024), the Framework to Identify Models of Disease (FIMD) for efficacy assessment in drug development (Ferreira et al., 2019), the Animal Model Quality Assessment (AMQA) framework supporting model selection and HBA (Storey et al., 2022), the Animal Model Framework (AMF), enabling predictive validity and performance (Valentin et al., 2009), GRADE4Animals, supporting decision-making on proceeding to clinical trials and the need for additional animal studies (Hooijmans et al., 2018), the SYstematic Review Centre for Laboratory animal Experimentation’s Risk of Bias (SYRCLE’s RoB) tool, providing a structured assessment of internal validity (Hooijmans et al., 2014), the Translatability score, enabling go/no-go decisions and guiding biomarker development (Wehling, 2009), the Replacement Checklist, implementing replacement due diligence (Dukes et al., 2025), and several other tools and frameworks.

However, the extent to which these tools are known by different preclinical research stakeholders remains uncertain. To explore this and identify potential strategies to facilitate their uptake and deployment, Humane World for Animals, through the Biomedical Research for the 21st Century (BioMed21) Collaboration, convened a workshop in 2025 (Pistollato et al., 2026) with tool and framework developers, researchers, members of ethics committees, representatives from government institutions, and industry professionals. Workshop participants generally agreed that currently available tools are rarely used in preclinical research by researchers, funding agencies, animal ethics committees, or scientific review boards. This limited uptake can be attributed to a combination of low awareness, insufficient systemic support, and unclear or contested validity and applicability of these tools.

As a complementary effort, BioMed21 launched a survey to explore levels of awareness within the preclinical research community regarding existing assessment tools, and to gather feedback on the most important criteria for assessing the translational relevance of animal models used in preclinical research.

Survey findings revealed moderate awareness of existing assessment tools, with 35% of participants reporting no prior knowledge of these tools. Key limitations identified included poor standardisation and reproducibility of assessment results, limited availability of high-quality comparative human data, lack of consensus on assessment criteria, and an over-reliance on expert opinion. Despite these limitations, participants perceived assessment tools as valuable for improving decision-making in model selection, supporting preclinical-to-clinical translation decisions, appraising animal model limitations, and enhancing the reproducibility of preclinical research. Across participants, human relevance consistently emerged as the most important criterion for assessing translational value. Here, we present survey findings and discuss a potential future strategy involving the development of a digital infrastructure to support the accessibility, navigation and integrated application of existing tools and frameworks.

## 2 Methodology

### 2.1 Questionnaire development and testing

The questionnaire was developed by the authors specifically for this study and was not based on a previously validated instrument. During its development, the authors reviewed previous surveys addressing researchers’ perspectives on experimental models and related aspects of biomedical research (Del Pace et al., 2022; Pistollato et al., 2024) which provided general methodological and contextual background but did not directly correspond to the aims or content of the present survey. The evaluation criteria included in Questions 3, 9 and 10 were developed specifically for this exploratory survey and were not derived from a single existing or validated assessment framework. They were intended to capture a broad range of potentially relevant considerations for assessing the translational value of preclinical models and to explore respondents’ perceptions of their relative importance. The lists were not intended to be exhaustive and were not established through a formal consensus-building or validation process. The draft questionnaire underwent two rounds of review involving researchers and subject-matter experts, including inputs from experts who had participated in a previous BioMed21 workshop on approaches for evaluating the translational value of animal models in preclinical research (Pistollato et al., 2026). The draft questionnaire was subsequently pilot-tested by five researchers to assess the clarity, relevance and comprehensibility of the questions and response options. The questionnaire was revised based on their feedback before dissemination. The original and administered language of the questionnaire was English. Test-retest reliability was not assessed as the questionnaire was developed as a descriptive and exploratory survey rather than as a theoretical or psychometric instrument or scale intended to measure a stable underlying construct. The individual questions and response options were therefore developed specifically to explore awareness, experience and opinions regarding tools and frameworks for assessing the translational value of preclinical models, rather than to develop or validate a measurement instrument.

The questionnaire was designed using the EU survey platform https://ec.europa.eu/eusurvey/runner/TranslationValueTools, all questions were displayed in one page, were presented in a fixed order, and adaptive questioning was not used (Supplementary File 1). The questionnaire comprised an introductory section outlining its overall objectives, three questions aimed at gathering information about survey participants, followed by ten multiple‐choice (for some questions respondents could indicate more than one option) or ordinal scale questions mainly aimed at:

i. exploring the level of awareness and gather feedbacks and opinions on tools and frameworks to assess translational relevance of animals models used in preclinical research;
ii. identifying strengths, limitations, efficiency, and feasibility of use of available tools; and.
iii. identifying perceived barriers to tools’ adoption in funding decisions and research evaluation processes.

For Q2 (*Were you already aware of the aforementioned tools/approaches to assess translational relevance of animals models?*), Q4 (*In your opinion, what are the potential limitations associated with tools that assess the translational value of animal models?*), and Q5 (*In what ways could tools that assess the translational value of animal models be beneficial to research institutions and/or funding bodies?*), participants were allowed to select more than one option. As a result, the combined percentages reported exceed 100%.

The reporting of the study followed both the CHERRIES guidelines (Eysenbach, 2004), which cover web-survey administration related aspects, and CROSS guidelines (Sharma et al., 2021), addressing the broader design and reporting of survey studies (Supplementary File 2, Tables S2.1 and S2.2).

### 2.2 Survey dissemination and study population

The survey targeted researchers and other professionals active in preclinical, nonclinical, translational and related biomedical research fields. A targeted, non-probability recruitment strategy was used. The link to the survey was initially posted by Humane World for Animals on social media through the BioMed21 LinkedIn channel (on June 17, 2025). In addition, potential participants were identified through corresponding-author email addresses retrieved from PubMed as previously described (Khalifa, 2019), using predefined combinations of affiliation and title/abstract search terms related to preclinical and translational research, drug development, animal models, non-animal methods and related topics. Searches were restricted to English-language publications published during the preceding five years. Duplicate email addresses were removed before dissemination. No formal eligibility screening questions or additional individual-level inclusion/exclusion criteria were applied. The complete search strategies, dates of dissemination and numbers of email addresses identified are provided in Supplementary File 3.

The survey invitation could also be shared with other professionals working in relevant areas of preclinical research, and no incentives were offered for participation. Given the use of multiple outreach approaches, it was not possible to determine the exact size of the target audience, defined as the number of potential respondents who encountered the survey link and recruitment message. Consequently, a response rate could not be calculated. Moreover, the questionnaire did not capture the recruitment channel through which individual respondents accessed the survey; therefore, the number of responses attributable to email invitations, social media dissemination, or secondary sharing of the survey link could not be determined. As of January 20, 2026, a total of 105 respondents had participated in the survey.

### 2.3 Ethical considerations and data management

This study was conducted in accordance with the Declaration of Helsinki. No prospective institutional ethics committee/IRB review or formal exemption determination was sought; the survey was organised by a non-governmental organisation (Humane World for Animals) and consisted of a voluntary, minimal-risk, non-interventional survey of adult professionals concerning their awareness and opinions regarding tools and frameworks for assessing translational value in preclinical research. Before proceeding, prospective participants were informed of the organisation conducting the survey, its purpose and scope, the expected completion time, and that their responses would remain anonymous. Participants were also provided with Humane World for Animals' Privacy Policy and were required to confirm that they had read and accepted its terms before accessing the survey (see Supplementary File 1). No separate written research-consent form was used; participants chose whether to proceed with and complete the survey after reviewing this information, with completion of the survey considered to indicate implied consent to participate.

No health, clinical or sensitive personal information was collected, and provision of identifying information was not required to participate in or complete the survey. At the end of the survey, participants who voluntarily indicated that they wished to be contacted regarding future activities could provide their name and email address. These optional details were associated with their corresponding survey responses in the exported dataset.

Following data collection, the dataset was exported from EUSurvey and stored on a secure Humane World for Animals-controlled drive, with access restricted to two authors of the study (FP and FF). The data will be retained for five years from data publication and subsequently securely deleted.

### 2.4 Data analysis and presentation

EUSurvey was configured so that all mandatory questions had to be answered before a questionnaire could be submitted, and respondents could review/change their answers only before final submission. The survey was configured to restrict participants to a single submission. Because partial/unsubmitted questionnaires were not retained by EUSurvey, the number of individuals who initiated but did not complete the survey was unavailable and a survey completion rate could not be calculated. Consequently, the analytical dataset comprised submitted questionnaires only, with no missing data for mandatory survey items, and no imputation of missing data was therefore required. No weighting, propensity-score adjustment or other correction for non-representativeness was performed.

Survey results were organised, analysed and plotted using the EU Survey Platform analytical tool and Microsoft Office Excel. Data were analysed descriptively using frequencies and percentages (calculated based on 105 participants). Additional exploratory descriptive analyses were conducted to examine responses to selected questions according to participants’ primary professional affiliation. Specifically, responses concerning perceived limitations of translational assessment tools (Q4 in the survey) and the applicability of assessment criteria to NAMs (Q6 in the survey) were cross-tabulated by affiliation category. Results were expressed as absolute frequencies and percentages within each subgroup (Suppl. File 2, Tables S2.3 and S2.4). Given the exploratory and descriptive nature of the survey and the limited sample size, no inferential statistical analyses or hypothesis testing were performed, and the findings were interpreted descriptively.

Free-text responses (not mandatory) were analysed using a descriptive narrative approach to complement the quantitative survey findings. Responses to each open-text question were reviewed and summarised according to the main points and perspectives expressed by respondents. Related observations were grouped for narrative presentation in the Results, while differing or contrasting perspectives were retained where relevant. No formal qualitative coding framework, thematic analysis, or quantitative assessment of the frequency of themes was undertaken. Accordingly, the synthesis was intended to provide a descriptive overview of the range of views expressed rather than to establish or quantify formally derived themes. All free-text responses are provided in Supplementary File 3.

## 3 Results

### 3.1 Information about survey participants

Out of the 105 participants who replied to the survey, 55% work in academia (n = 58), 14% for government or public institutions (n = 15), 10% in clinics of hospitals (n = 10), and equal 8% (n = 8) in contract research organisations (CROs) and non-governmental organisations (NGOs), and 5% (n = 5) in industry (Fig. 1A). Forty-five percent of participants are senior scientists (n = 47), 26% are group leaders (n = 27), 9% clinicians (n = 9), 6% post-docs (n = 6), 5% PhD students (n = 5), and 4% regulatory decision makers (n = 4) (Fig. 1B).

**Fig. 1:**
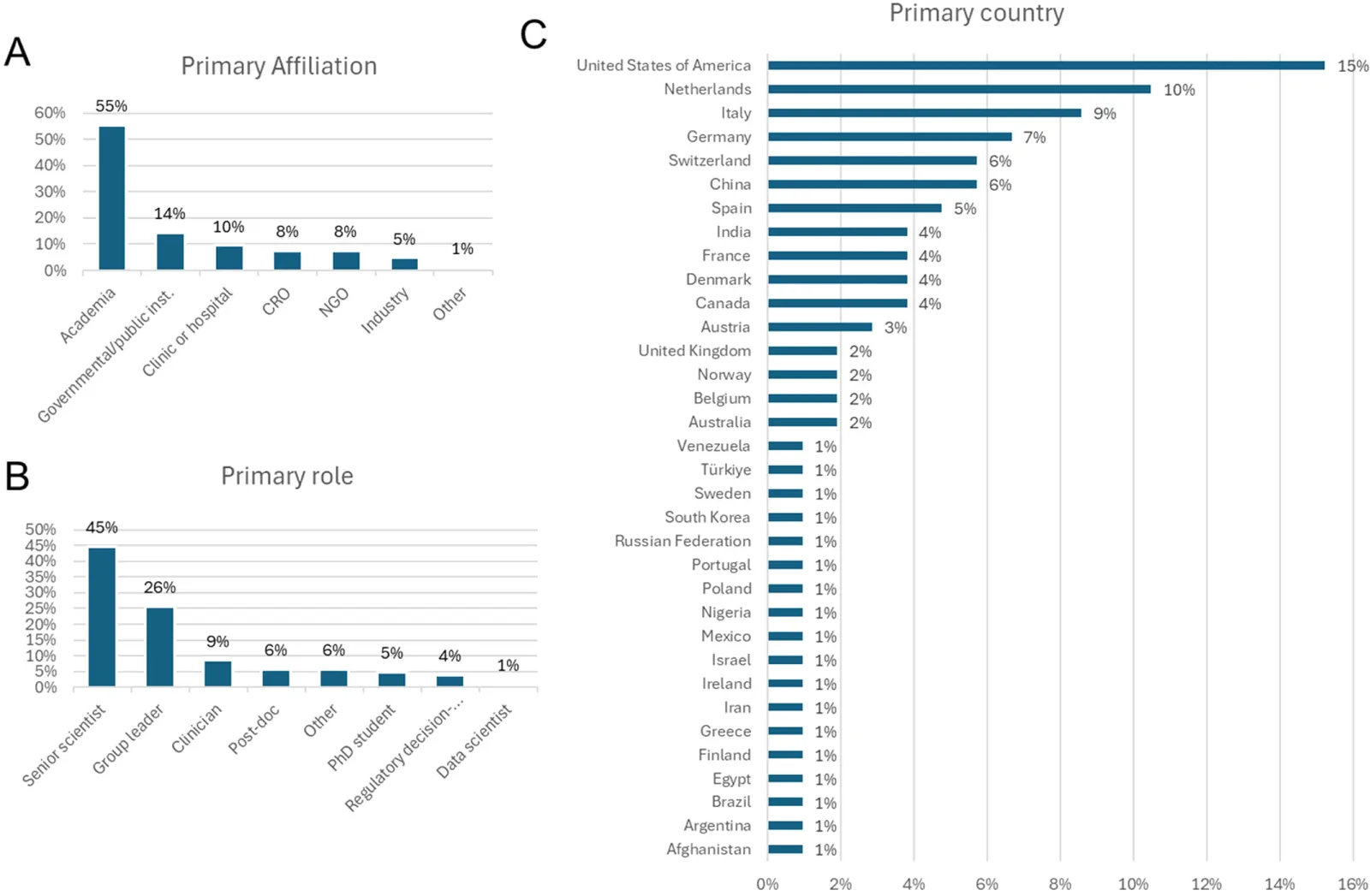
Survey participants’ primary affiliations (A), role (B), and country (C). Percentage of respondents are reported.

When considering participants’ primary country, 15% of them work in the United States (n = 16), 10% in the Netherlands (n = 11), 9% in Italy (n = 9), 7% in Germany (n = 7), an equal 6% in Switzerland and China (n = 6), and 5% in Spain (n = 5). Other regional affiliations included India, France, Denmark, Canada and other countries, as reported in Fig. 1C.

### 3.2 Respondents’ level of awareness of existing tools and frameworks

When asked about the importance of having tools to assess translational relevance of animal models, 93% of participants considered this important, with approximately three-quarters of respondents (74%, n = 78) rating it as highly important, while only 2% considered it not important (Fig. 2A).

**Fig. 2:**
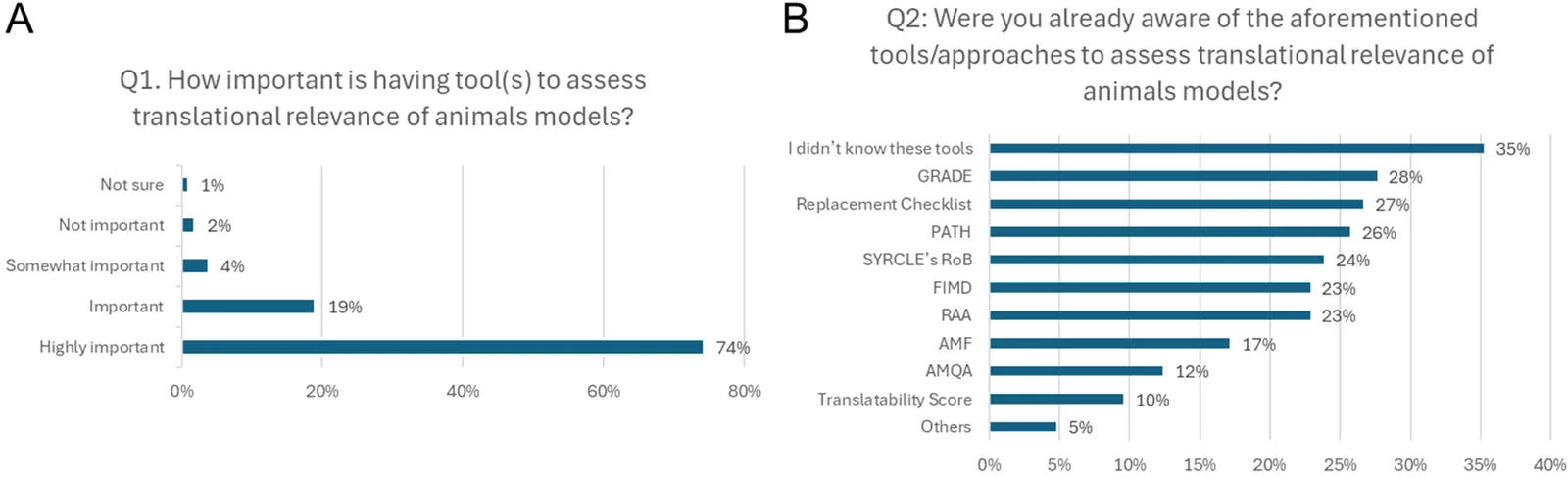
Importance of having tools to assess translatability (A) and general awareness of available tools (B). Reported values represent percentages of participants. In B, participants were asked to select at least one option, which explains why the combined percentages exceed 100%.

In another question, examples of tools to assess translatability, such as PATH, RAA, FIMD, AMQA, AMF, or frameworks to evaluate the risk of bias (e.g. SYRCLE’s RoB), rate the certainty in the evidence (e.g. GRADE), or evaluate animal replacement opportunities (e.g. Replacement Checklist), were provided. Participants were asked whether they were already aware of such tools; 35% of respondents (n = 37) declared they were not aware of any (Fig. 2B); 28% knew about GRADE (n = 29), 27% heard about the Replacement checklist (n = 28), 26% about PATH (n = 27), 24% about SYRCLE’s RoB (n = 25), and an equal 23% (n = 24) knew about FIMD and RAA. Other tools were indicated by participant as reported in Fig. 2B.

### 3.3 Most important criteria to evaluate the translational value of preclinical animals models

Next, we asked participants to rate as either ‘not important’, ‘somewhat important’, ‘important’ or ‘very important’ or to indicate ‘not sure’ in case they had no opinion a list of criteria to be considered when evaluating the translational value of animals models in preclinical research. Among the criteria rated as ‘very important’, relevance of the model to human physiology (77%, n = 81), reproducibility and robustness of the results in the model (74%, n = 78), predictive validity for clinical outcomes (69%, n = 72), ethical issues and severity of procedures (62%, n = 65), and mechanistic similarity to human biology (60%, n = 63) were the five most selected options, in this order (light blue bars, Fig. 3).

**Fig. 3:**
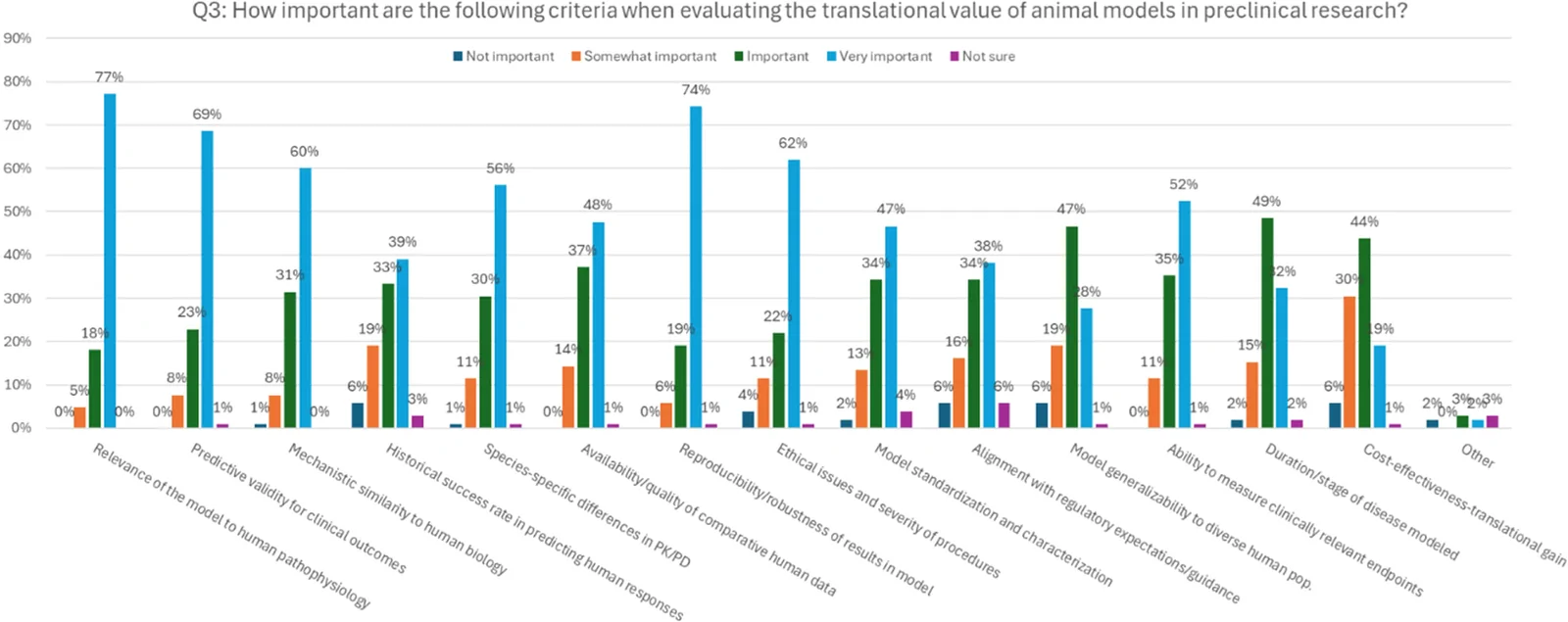
**Criteria to evaluate the translational value of preclinical animal research**. Participants were asked to rate the level of importance of a list of criteria potential suitable for assessing the translational value of preclinical animal models (reported in the x axis). Each criterion could be scored as either not important (dark blue), somewhat important (orange), important(green), very important (light blue), or not sure (purple). Reported values represent percentages of participants.

Among the criteria deemed ‘important’ the five most frequently selected were duration and stage of disease modeled (49%, n = 51), model generalisability to diverse human populations (47%, n = 49), cost effectiveness relative to translation gain (44%, n = 46), availability and quality of comparative human data (37%, n = 39), while an equal 34% (n = 36) selected model standardisation and characterisation level and alignment with regulatory expectations or guidance (dark green bars, Fig. 3).

The additional free-text comments provided further perspectives on criteria for evaluating translational value. One respondent emphasised that phenotypic resemblance to human disease does not necessarily imply predictive value and advocated direct comparison between outcomes observed in animal models and corresponding human outcomes, while also raising concerns about scientific inertia sustaining the use of established models with limited predictive relevance. Another respondent cautioned against expecting individual models to meet all assessment criteria or to be broadly generalisable, emphasising instead that model relevance should be evaluated in relation to the specific research question. Other proposed considerations included the use of endpoints already accepted in regulatory contexts and the acceptability of evidence in high-quality publications (Supplementary File 3).

### 3.4 Perceived tools’ limitations and suitability

About half of survey participants (n = 53) believe that currently available tools and frameworks are mainly limited by poor standardisation and reproducibility of assessment results, as well as the limited availability of high-quality comparative human data (n = 52), and the overall lack of consensus on criteria to include (n = 52) (Fig. 4A).

**Fig. 4:**
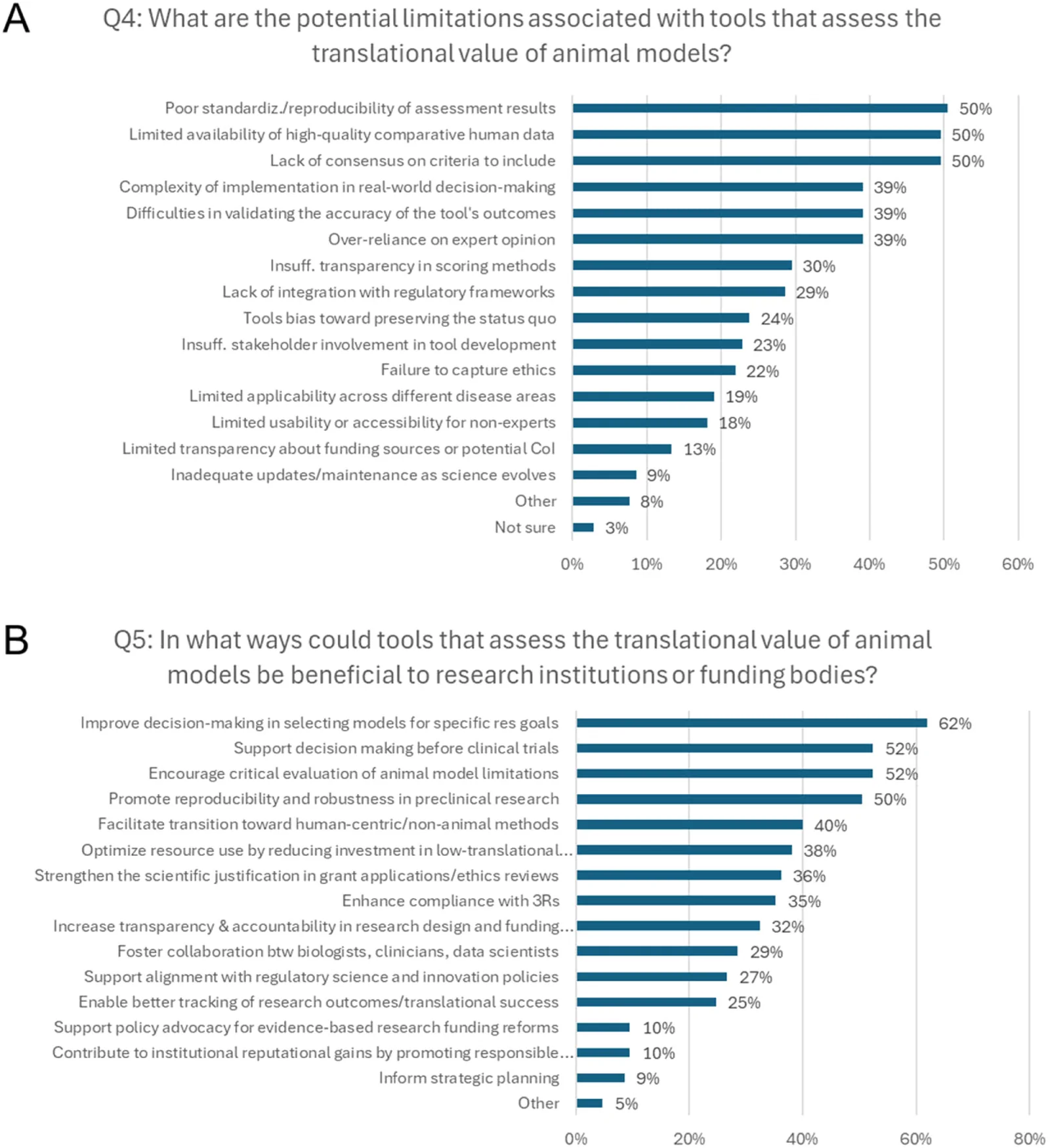
**Perceived potential tools’ limitations (A) and potential benefits associated with their use (B).** Reported values represent percentages of participants. Participants were asked to select at least one option, which explains why the combined percentages exceed 100%.

In addition, an equal 39% (n = 41) think that complexity of implementing these tools in real-world decision-making, as well as difficulties in validating the accuracy of the tool's outcomes, and the over-reliance on expert opinion are potential limitations associated with tools that assess the translational value of animal models (Fig. 4A).

Additional free-text comments identified a range of potential limitations or broader considerations. Individual respondents pointed to the lack of systematic review or evidence synthesis as a mandatory component of assessment processes, insufficient incentives to move beyond traditional approaches, and limited expertise and funding. Other comments concerned the selection of animal models that may not adequately represent the human situation, insufficient consideration of pharmacokinetic differences between preclinical species and humans, and limited awareness of how experimental context and study conditions may influence data interpretation. Differing perspectives were also expressed regarding the role of assessment tools and alternative approaches: one respondent considered the use of valid animal models more important than assessment tools themselves, while another cautioned against framing alternative test systems simply as replacements for animal experiments and instead advocated their development with the aim of making animal experiments redundant (Supplementary File 3).

An additional exploratory analysis was conducted to examine whether perceptions of the limitations of translational assessment tools differed according to respondents’ primary professional affiliation (Supplementary File 2, Table S2.3). Some descriptive differences were observed across stakeholder groups. Poor standardisation or reproducibility of assessment results was selected by 80% of industry respondents (n = 4/5), compared with 60% of respondents from governmental/public institutions (n = 9/15), 48% of academic respondents (n = 28/58), and 30% of clinic/hospital respondents (n = 3/10). Lack of consensus on assessment criteria was more frequently selected by respondents from governmental/public institutions (67%, n = 10/15), CROs (63%, n = 5/8), and clinic/hospital representatives (60%, n = 6/10), than by academic respondents (43%, n = 25/58) or NGO representatives (38%, n = 3/8). Complexity of implementation in real-world decision-making was selected by 63% of respondents from NGOs (n = 5/8) and 60% from industry (n = 3/5), compared with 40% of respondents from government/public (n = 6/15) and 34% from academia (n = 20/58). Difficulties in validating the accuracy of assessment outcomes were selected by 41% of academic respondents (n = 24/58) and 40% of government/public respondents (n = 6/15), compared with 20% of industry representatives (n = 1/5). In addition, lack of integration with regulatory or policy frameworks was most frequently selected by industry respondents (60%, n = 3/5) and government/public institute representatives (47%, n = 7/15), than those from academia (19%, n = 11/58). These findings are descriptive and should be interpreted cautiously given the small and uneven subgroup sizes, particularly for industry, NGOs and CROs.

When asked about the potential benefits of translational assessment tools for research institutions and/or funding bodies, 62% of respondents (n = 65) indicated that they could help improve decision-making in selecting models for specific research goals, an equal proportion (52%, n = 55) considered that such tools could support decision making before clinical trials, and the critical evaluation of animal model limitations, whilst 50% (n = 53) indicated that they can help increase reproducibility and robustness in preclinical research (Fig. 4B).

When asked about the applicability of scoring criteria that were originally developed to assess the translational value of preclinical animal models to non-animal methods, nearly half of participants (49%, n = 51) indicated that these criteria could be partially applicable to non-animal methods if appropriate adaptations were made. A further 26% (n = 27) were unsure, while 18% (n = 19) felt that non-animal methods would require completely different assessment strategies. Only 8% (n = 8) believed that the same criteria could be fully applicable to NAMs (Fig. 5).

**Fig. 5:**
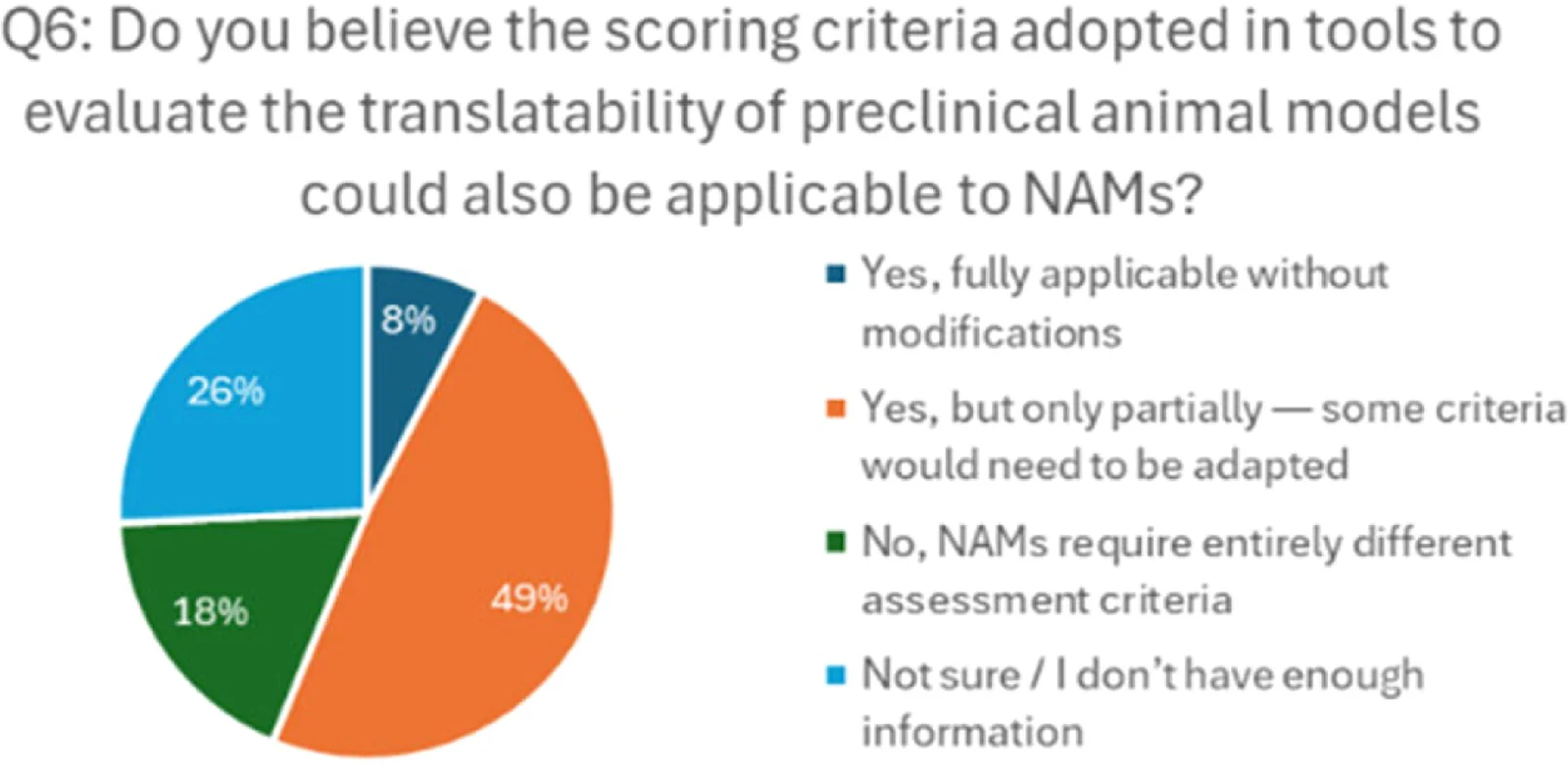
**Applicability to non-animal methods (NAMs) of criteria used to assess translational value of preclinical animal models**. Reported values represent percentages of participants.

When considering distributions of replies across respondents’ primary affiliations, some differences emerged (Supplementary File 2, Table S2.4). In particular, all 8 NGO representatives (100%) considered that existing criteria could be partially applied to NAMs, with adaptations; 60% (9/15) of governmental/public institution representatives also supported partial applicability, compared with 40% (23/58) of academic respondents. Responses from CRO representatives were more divided, with 38% (3/8) who considered that NAMs require entirely different assessment criteria, while 25% (2/8) supported partial applicability. Concerning academia representatives, nearly one-third (33%, 19/58) selected “not sure,” compared with 13% (2/15) of governmental/public institution respondents.

### 3.5 Possible sources of bias in preclinical animal research

Participants were next asked to indicate their views on the most important sources of bias in preclinical research. A list of potential issues was provided (shown on the x-axis in Fig. 6), and participants were asked to rate their level of agreement with each as a potential source of bias. Notably, 60% of participants (n = 63) completely agreed that publication bias (i.e., the selective publication of positive results) represents an important source of bias, paralleled by 46% (n = 48) strongly agreeing that incomplete reporting of negative or null results is a significant issue. Overinterpretation or overselling of results to suggest human relevance was identified as a significant issue by 45% of participants (n = 47), and 40% (n = 42) highlighted selective outcome reporting as an additional relevant source of bias. Furthermore, 39% (n = 41) indicated poor or inconsistent study design as a relevant issue (light blue bars, Fig. 6).

**Fig. 6:**
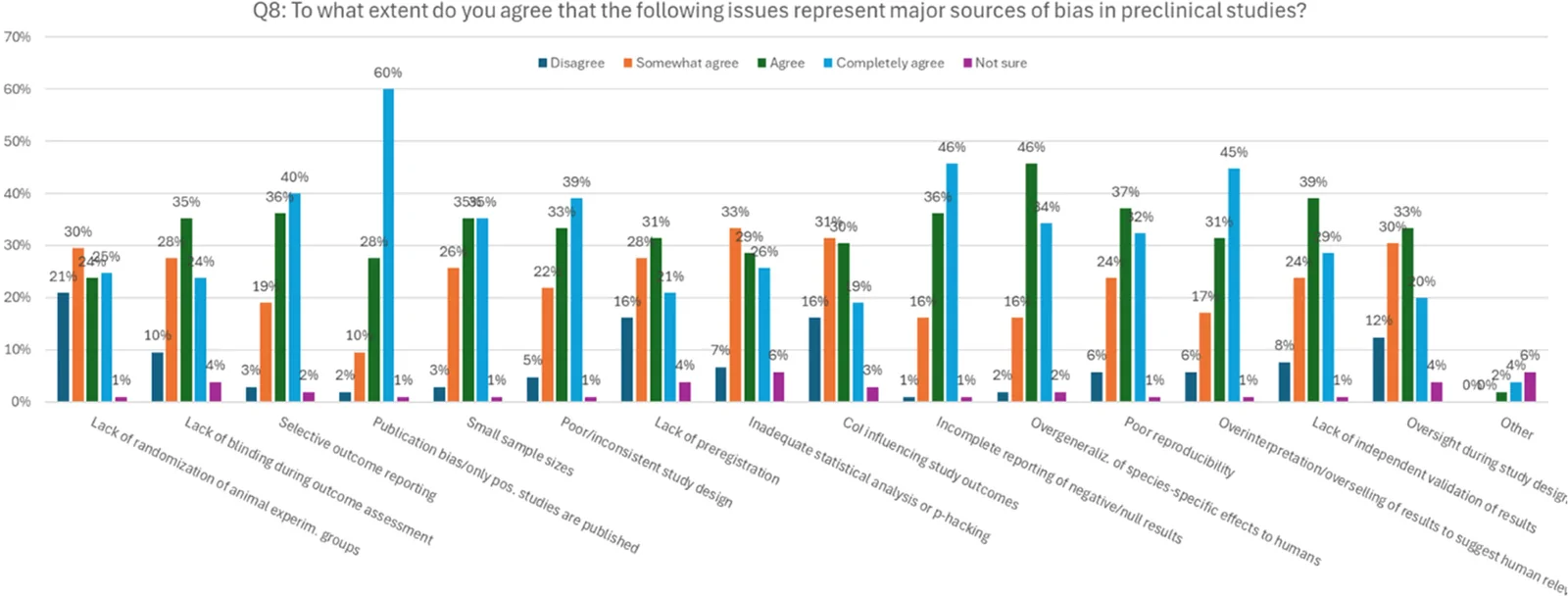
**Sources of bias in preclinical animal research.** Participants were asked to express their level of agreement – as either completely agree (light blue), agree (green), somewhat agree (orange), disagree (dark blue), or not sure (purple) – with a provided list of issues as potential sources of bias. Reported values represent percentages of participants.

In addition, 46% of respondents (n = 48) generally agreed that the overgeneralisation of species-specific effects to humans also represents an important source of bias (green bars, Fig. 6).

Additional free-text comments identified further considerations regarding potential sources of bias in preclinical studies. Individual respondents raised concerns about the relevance and quality of animal models for representing human disease, including the limited availability of suitable models for psychiatric disorders and aspects such as model standardisation, repeatability and stability. Lack of knowledge of animal anatomy, physiology and pathology was also identified as a potential source of bias. Other individual comments highlighted ambiguity in the meaning of “bias” itself and the fragmentation and lack of systematically connected data. One respondent also suggested that non-GLP pharmacological proof-of-concept studies may be particularly susceptible to bias or misinterpretation, especially when conducted in academic settings with limited regulatory experience. Conversely, another respondent cautioned that the potential sources of bias listed in the questionnaire are not specific to animal research and may apply more broadly across different types of research (Supplementary File 3).

### 3.6 What need to be scored to predict translational success of preclinical research

Participants were asked to rate the importance of different aspects that could be scored to predict the translational success of a preclinical model, whether animal or non-animal.

Biological relevance to human pathophysiology (64%, n = 67), predictive validity (e.g., efficacy, toxicity outcomes in humans) (59%, n = 62), inter-laboratory and inter-study reproducibility (53%, n = 56), statistical method and experimental design robustness (52%, n = 55), and transparency of reporting and data availability (49%, n = 51) were deemed as ‘very important’ aspects to consider (light blue bars, Fig. 7).

**Fig. 7:**
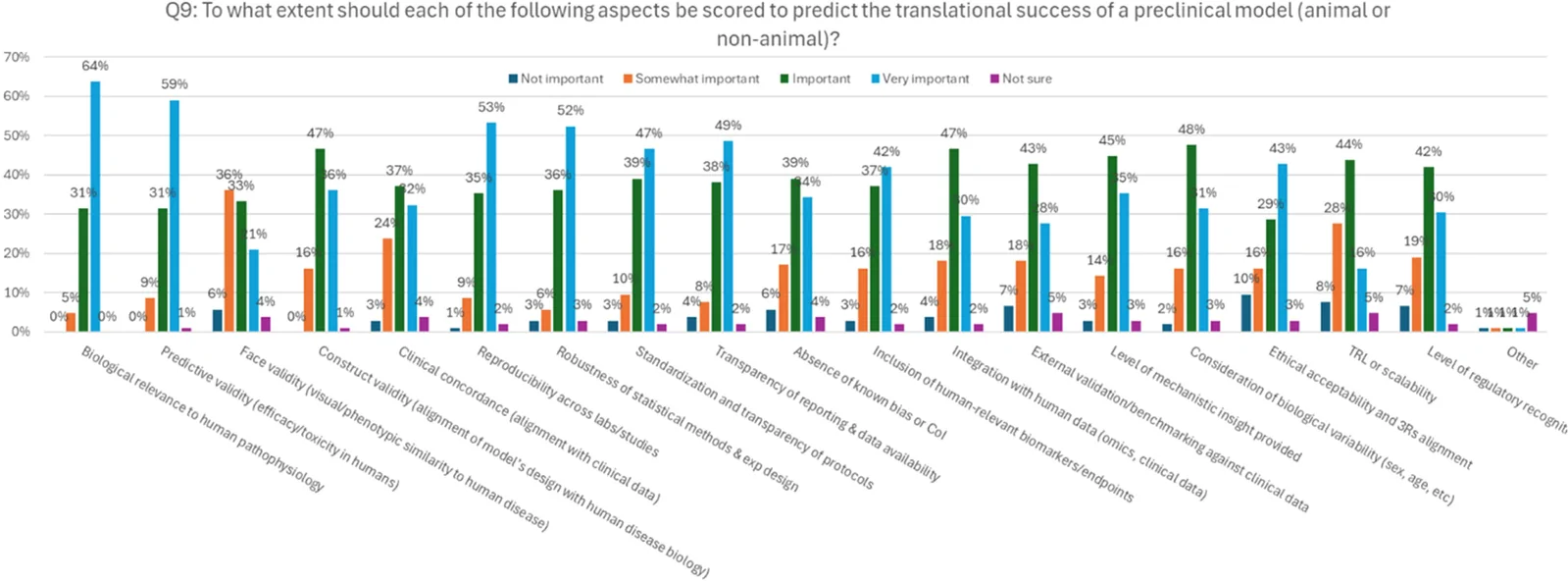
**Aspects to be scored to predict translational success of preclinical research.** Participants were asked to rate each of the provided aspects as very important (light blue), important (green), somewhat important (orange), not important (dark blue), or not sure (purple). Values are reported as percentages of participants.

Among the aspects considered ‘important’, consideration of biological variability (sex, age, genetics, comorbidities) (48%, n = 50), construct validity (alignment of model’s design with human disease biology) (47%, n = 49), integration with human data (e.g., omics, clinical data) (47%, n = 49), level of mechanistic insight provided (45%, n = 47), and technical readiness level or scalability (44%, n = 46) were the most selected in this order (green bars, Fig. 7).

### 3.7 Criteria to consider when evaluating animal replacement opportunities

Furthermore, we asked participants to express their views on the most relevant criteria to consider when evaluating animal replacement strategies. Among those deemed as ‘very important’ an equal 60% of participants (n = 63) considered the biological and mechanistic relevance to human disease compared to animal models, as well as the predictive accuracy for human outcomes relative to animal model performance. Reproducibility and robustness across laboratories versus variability in animal studies was considered as ‘very important’ by 57% of respondents (n = 60), whilst transparency and completeness of protocols and reporting for NAMs compared to animal studies by 49% (n = 51). Moreover, 46% (n = 48) rated as ‘very important’ the independent validation and benchmarking of NAMs against both animal data and clinical outcomes (light blue bars, Fig. 8).

**Fig. 8:**
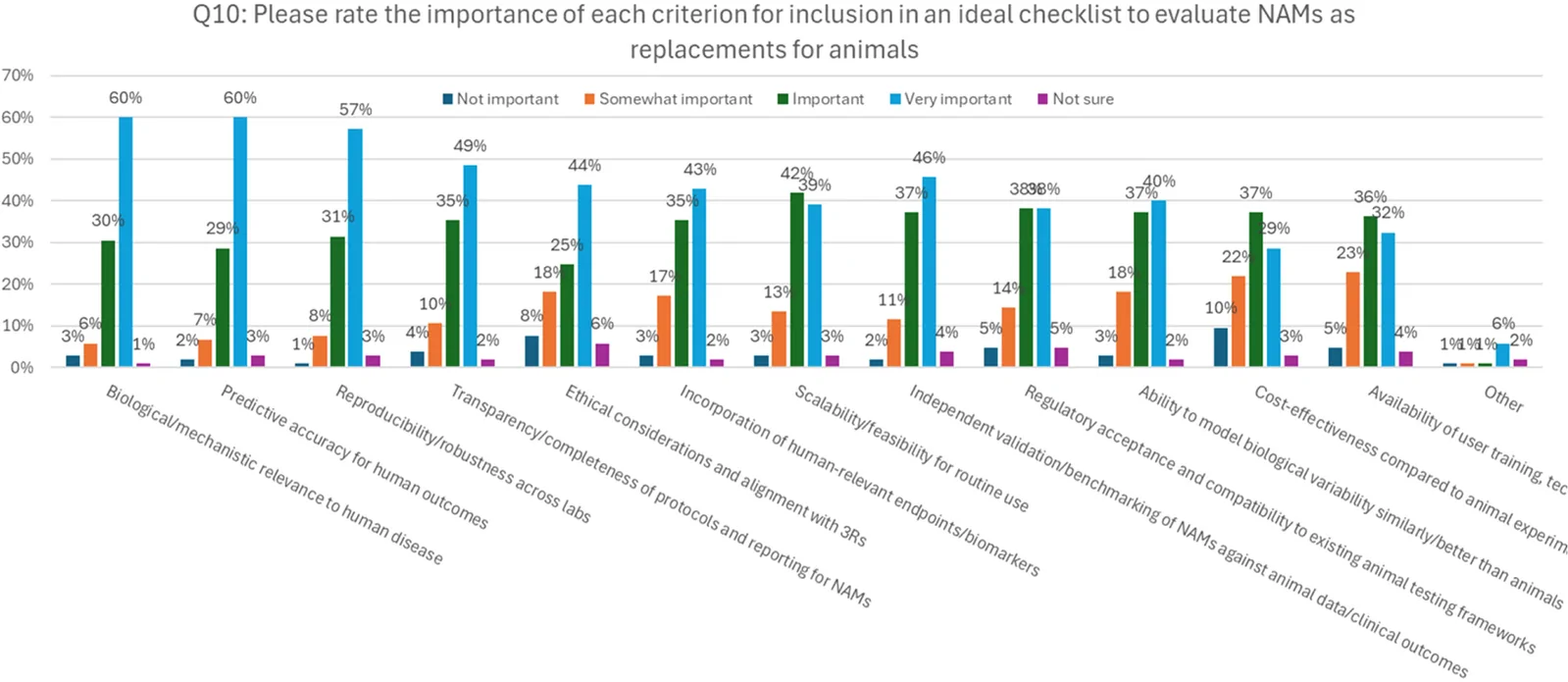
**Criteria to consider when evaluating animal replacement opportunities.** Participants were asked to rate the importance – either as very important (light blue), important (green), somewhat important (orange), not important (dark blue), or not sure (purple) – of a list of provided criteria for inclusion in an ideal checklist to evaluate NAMs as replacements for animals. Values are reported as percentages of participants.

Among the criteria rated as ‘important’, 42% of participants (n = 44) opted for scalability and practical feasibility for routine use in comparison to animal testing. Regulatory acceptance and compatibility relative to existing animal testing frameworks was considered as important by 38% of respondents (n = 40), while 37% (n = 39) think that cost-effectiveness and resource requirements compared to animal experiments play an important role (green bars, Fig. 8). Other criteria rated as ‘important’ are shown in Fig. 8 (green bars).

Additional free-text comments reflected differing perspectives on the criteria for comparing NAMs with animal models (Supplementary File 3). One respondent questioned the premise of direct comparison, arguing that NAMs should instead be evaluated according to their ability to inform human clinical relevance, while another emphasised that both animal and non-animal models have inherent limitations that should be recognised when evaluating their performance. Other individual comments highlighted the importance of anticipating potential interfering effects, accounting for variability and whole-organism context, and considering what reference should be used when validating NAMs. One respondent also emphasised the need to consider cell-type- and organ-specific functional limitations of in vitro systems. Overall, the limited free-text responses reflected diverse views on whether direct comparison between NAMs and animal models is appropriate and on the criteria that should inform such evaluations.

Finally, more than half of participants (53%, n = 56) expressed interest in this initiative and were willing to be further recontacted for follow-up activities.

## 4 Discussion

### 4.1 General awareness of available tools and perceived barriers to their use

Several tools and frameworks have been developed over the past two decades to strengthen scientific due diligence in the assessment of preclinical research and its translatability to humans (Gurusamy et al., 2021; Kimmelman et al., 2024), to appraise animal model performance in efficacy testing (Ferreira et al., 2019; Storey et al., 2022), to support predictive validity (Valentin et al., 2009), to evaluate risk of bias and internal validity (Hooijmans et al., 2014), to support HBA (Azilagbetor et al., 2024), to inform go/no-go decisions (Hooijmans et al., 2018; Wehling, 2009), or to identify opportunities for animal replacement (Dukes et al., 2025).

In an effort to examine to what extent such tools and frameworks are known by researchers and other preclinical stakeholders, we launched a survey as a complementary scoping exercise to previous BioMed21 initiatives aimed at stimulating debate on translational relevance across multistakeholder levels (Pistollato et al., 2026). The vast majority of survey participants (93%) believe that the availability of assessment tools is either important or highly important. However, despite the fact that these tools and frameworks are publicly available, our survey findings indicate that 35% of participants were unaware of them prior to the survey. This observation is corroborated by findings from the BioMed21 workshop (Pistollato et al., 2026), where participants confirmed that the overall use of such tools remains limited. According to workshop participants, perceived barriers to tool adoption include the lack of regulatory mandates, weak incentives, and limited awareness and training, further reinforced by cultural and institutional inertia. In addition, the absence of large-scale validation of their impact on translational assessment may further reduce their perceived value. Moreover, survey results also indicate that poor standardisation and reproducibility of assessment results, as well as the limited availability of high-quality comparative human data, the lack of consensus on assessment criteria, and the need to rely on expert judgment all may contribute to limiting the broader adoption of existing tools and frameworks.

### 4.2 Perceived utility of assessment tools and priority criteria for translatability assessment

Survey participants indicated that these assessment tools could support decision-making in model selection for specific research goals, facilitate decision making prior to entering clinical trials, enable a more systematic appraisal of animal model limitations, and enhance the reproducibility of preclinical research.

These aspect are particularly relevant given that animal studies frequently undergo rapid, and at times superficial, scrutiny and approval (Azilagbetor et al., 2024; Busquet et al., 2020), while persistent challenges related to preclinical research reproducibility can substantially hinder drug development and lead to inefficient use of resources (Pusztai et al., 2013; Reynolds, 2022).

Moreover, approximately three-quarters of survey participants identified relevance to human physiology as the most relevant criterion for assessing the translational value of preclinical animal models. Other desirable criteria included reproducibility and robustness of the results, predictive validity for clinical outcomes, and consideration of ethical aspects and procedural severity.

While human relevance is often defined by the origin of the cellular, tissue, or organ system used, demonstrating predictive validity, that is, the ability of a model to reliably recapitulate clinically meaningful endpoints, remains a key criterion for evaluating the suitability of preclinical models (Gurusamy et al., 2021). In this context, the availability and accessibility of high-quality clinical datasets are of paramount importance for qualifying and benchmarking preclinical models. However, a substantial proportion of these data is never made publicly available (Fortunato et al., 2018). In addition, clinical data fragmentation, driven by non-interoperable systems, proprietary silos, and regulatory, legal, or commercial barriers (ChapsVision, 2026; Xafis and Labude, 2019), may further limit the ability to perform robust comparative analyses of model performance. This challenge is particularly pronounced for academic researchers and contract research organisations, who are often at the forefront of developing and testing innovative NAMs.

### 4.3 Sources of bias and animal replacement due diligence

When reflecting on potential sources of bias in preclinical research, participants most frequently identified selective publication of positive findings, overselling of results to imply human relevance, selective outcome reporting, and poor or inconsistent study design as major concerns. These factors may systematically distort the evidence base, inflate perceived translational value causing spurious, non-replicable findings being interpreted as robust evidence (Mlinarić et al., 2017; Sharma and Verma, 2019; Smith and Noble, 2025), thereby complicating objective assessment of model performance (Hooijmans et al., 2018, 2014; Krauth et al., 2013).

With respect to animal replacement due diligence, respondents prioritised criteria emphasising human biological relevance and comparative predictive performance, reflecting a clear shift toward outcome-oriented and human-centred evaluation standards. In particular, biological and mechanistic relevance to human disease, predictive accuracy for human outcomes relative to animal model performance, and inter-laboratory reproducibility were considered especially important. These priorities underscore the need for robustness, transferability, and consistency across independent settings. Such criteria suggest a growing expectation that NAMs should not only be scientifically innovative, but also demonstrably reliable and informative for human decision-making, as discussed e.g., in the context of Artificial Intelligence (AI) technologies (Kinney et al., 2024). NAMs judged to be reliable and human-relevant may therefore be implemented in industry settings to inform business and development decisions ahead of, or alongside, their progression toward formal regulatory acceptance (Parish et al., 2020).

Participants also highlighted the importance of transparency and completeness of protocols and reporting for both NAMs and animal studies, recognising that inadequate documentation can hinder reproducibility, limit independent assessment, and obscure sources of bias (Jones and Martin, 2021), as also discussed above. In addition, respondents raised different perspectives regarding the validation and benchmarking of NAMs, including the importance of assessing their relevance to human clinical outcomes and questions around the appropriateness of using animal data as a reference for their validation. This reflects general awareness that confidence in animal replacement strategies depends on credible, comparative evidence that links model outputs to human-relevant endpoints (Mirlohi et al., 2025).

### 4.4 Study limitations

This study has limitations. Although the survey had a global scope, the geographical distribution of respondents was uneven, with 41% originating from four countries (the United States, the Netherlands, Italy and Germany). As the questionnaire did not capture the recruitment channel through which individual respondents accessed the survey, the reasons for this imbalance cannot be determined. It may partly reflect the geographical distribution of researchers identified through the PubMed-based recruitment strategy, as well as the reach of the BioMed21 LinkedIn channel, the authors' professional networks and secondary dissemination of the survey. More broadly, the targeted, non-probability recruitment approach may have introduced recruitment bias and limits the representativeness of the sample.

As participation was voluntary, self-selection and non-response bias cannot be excluded. In particular, recruitment through the BioMed21 LinkedIn channel and PubMed-based identification of researchers may have disproportionately reached individuals with a pre-existing interest in translational relevance, research methodology, or the assessment tools covered by the survey. Such individuals may also have been more likely to participate, potentially resulting in an overrepresentation of respondents with greater awareness of or engagement with translational assessment approaches. This is particularly relevant when interpreting the finding that 35% of respondents reported never having heard of the assessment tools covered by the survey. This percentage should not be interpreted as an estimate of the prevalence of unfamiliarity with these tools across the broader preclinical research community. Indeed, if researchers already interested in translational assessment were more likely to participate, the proportion of researchers unfamiliar with these tools in the wider community could be substantially higher than that observed in our sample. However, because information on non-respondents was unavailable, the magnitude and direction of this potential bias cannot be established.

A conventional overall response rate could not be reliably calculated because the survey was disseminated through multiple channels, including email, LinkedIn and secondary sharing, and the recruitment channel of individual respondents was not recorded. Although 23,090 unique email addresses were targeted, approximately 10–25% of emails were returned as undeliverable, and it was not possible to determine how many submitted responses originated from the email invitations. Moreover, information on non-respondents was unavailable, precluding comparison between respondents and non-respondents. Together with the relatively limited sample size, these factors further limit the generalisability of the findings to the broader global preclinical research community.

Finally, the study was designed as an exploratory and descriptive survey rather than to test predefined hypotheses. Accordingly, analyses were limited to descriptive statistics, and no inferential statistical analyses were performed. The findings should therefore be interpreted as reflecting the awareness, experiences and opinions of the surveyed participants rather than as population-level estimates. Along the same line, the exploratory analyses stratified by professional affiliation should also be interpreted cautiously, as the relatively small and unevenly distributed stakeholder subgroups limit the robustness of comparisons. Any apparent differences between groups may reflect sampling variability or differences in recruitment and participation rather than systematic differences in stakeholder perspectives.

Despite these limitations, some of the perspectives expressed by survey respondents are consistent with observations and recommendations from the previous BioMed21 workshop (Pistollato et al., 2026).

### 4.5 Conclusions and future directions

Study findings indicate variable and, for several existing tools, limited awareness among surveyed participants, while also identifying perceived strengths, limitations and potential barriers that may influence their broader uptake. Further work is needed to determine the extent to which existing translational assessment tools are currently used in research and decision-making and to evaluate strategies that could facilitate their wider adoption.

Beyond the findings of the present survey, recent analysis of the landscape of translational assessment instruments has highlighted fragmentation and limited interoperability across existing approaches, which generally address distinct aspects of the translational process and are not designed to operate within an integrated evidence-to-decision workflow (Straccia et al., 2026). Some approaches also require resource-intensive evidence synthesis and specialised expertise, which may present practical challenges to their routine application. These observations, together with the perceived barriers identified by respondents in the present survey, suggest that improving accessibility, navigation and interoperability across existing tools could represent a useful area for future development.

One potential direction for future work would be to explore whether AI-driven approaches, including advanced natural language processing (NLP), could support more integrated and efficient navigation and application of existing tools and frameworks. Rather than replacing individual assessment instruments or expert judgement, such an approach could potentially function as a decision-support and navigation layer, helping users identify appropriate tools according to the research question, context of use and applicability domain. For example, it could guide users towards existing approaches for identifying animal replacement opportunities, evaluating the translational relevance of animal models, assessing risk of bias, or conducting harm-benefit analyses.

As recently proposed (Straccia et al., 2026), such an AI-supported workflow could potentially serve different stakeholders, including academic researchers designing preclinical studies, funding bodies and research evaluators assessing proposals, and institutional ethics committees and competent authorities reviewing projects involving live animals. Potential benefits could include improved navigation across currently fragmented approaches, greater consistency and auditability of evidence assessment, and enhanced reuse of preclinical evidence. However, these potential benefits were not evaluated in the present survey and should be regarded as hypotheses for future development and testing. Prospective evaluation would be needed to determine whether such an approach improves usability, reduces variability or subjectivity in evidence assessment, facilitates uptake of existing tools, or ultimately contributes to more reliable and human-relevant biomedical research.

## Ethics declaration

The authors have not obtained written or verbal informed consent from participants or their legal representatives because participation in this voluntary, minimal-risk survey of adult professionals was based on implied consent. Before proceeding, participants were provided with information about the purpose and expected duration of the survey and were required to acknowledge the applicable privacy statement. Participants then chose voluntarily whether to complete and submit the questionnaire, and submission of the questionnaire was considered to indicate consent to participate. No patients, clinical interventions or biological samples were involved, and no health or other sensitive personal data were collected. Survey findings are reported in aggregate, and no information intended to identify individual participants is included in the manuscript.

This study was performed in compliance with relevant laws, regulatory frameworks and guidelines where the research took place. Ethics committee approval was not required under relevant laws and institutional guidelines. Formal ethics committee review was not sought for this study. The survey was conducted by Humane World for Animals, a non-governmental organisation, as a voluntary, minimal-risk, non-interventional survey of adult professionals responding in their professional capacity. The survey did not involve patients, clinical interventions, biological samples, or the collection of health or other sensitive personal data. Participants were provided with information about the purpose of the survey and the applicable privacy statement before participation and voluntarily completed the questionnaire. On this basis, formal ethics committee review was considered not required.

## Declaration of generative AI and AI-assisted technologies in the manuscript preparation process

During the preparation of this work, the authors used ChatGPT (OpenAI) to support language editing and improve the clarity, readability and structure of the manuscript. After using this tool, the authors reviewed and edited the content as needed and take full responsibility for the content of the published article.

## Funding

This activity was supported by Lush Ltd. through a grant to Humane World for Animals.

## CRediT authorship contribution statement

**Francesca Pistollato:** Writing – review & editing, Writing – original draft, Visualization, Supervision, Project administration, Methodology, Formal analysis, Conceptualization. **Ignacio J. Tripodi:** Writing – review & editing, Writing – original draft. **Fabia Furtmann:** Writing – review & editing, Writing – original draft, Formal analysis, Data curation. **Marco Straccia:** Writing – review & editing, Writing – original draft, Conceptualization. **Helder Constantino:** Writing – review & editing, Writing – original draft, Project administration.

## Declaration of competing interest

The authors declare that they have no known competing financial interests or personal relationships that could have appeared to influence the work reported in this paper.

## Data Availability

Data will be made available on request.
