# Supplementary material for: Awareness and perspectives of translational assessment tools in preclinical research: insights from a survey
Source: NAM J. 2026 Oct 13;2:100146. doi: 10.1016/j.namjnl.2026.100146 (PMC13696333; doi:10.1016/j.namjnl.2026.100146)
Supplement: Supplementary file 1 [file mmc1.pdf]

# Tools to assess translational value of animal models in preclinical research

Fields marked with * are mandatory.

## Introduction

In today's evolving research landscape—shaped by growing global support for a transition toward human-centric approaches—it is becoming increasingly important to objectively assess the scientific relevance and merit of studies, particularly those involving animal models.

On behalf of the Biomedical Research for the 21st Century (BioMed21) Collaboration, Humane World for Animals has launched the following survey aimed to understand the level of awareness and gather feedbacks and opinions on available tools and frameworks to assess translational relevance of animals models used in preclinical research.

Such tools include, for example, the Clinical relevance assessment of animal preclinical research (RAA) tool, the PATH approach, the FIMD framework to identify optimal animal models for efficacy assessment in drug development, the Animal Model Quality Assessment framework, the Animal Model Framework (AMF), GRADE (Grading of Recommendations, Assessment, Development, and Evaluation), SYRCLE's risk of bias tool for animal studies, the Translatability score, and the Replacement Checklist.

The survey aims to assess overall awareness of these tools, as well as to identify their strengths, limitations, efficiency, and feasibility of use. It will also help uncover potential barriers to their adoption in funding decisions and research evaluation processes.

The survey will take approximately 15 minutes to complete, and please note that all responses will remain anonymous.

Feel free to share the survey link with representatives of preclinical research organizations who may be interested in these topics.

Privacy statement
Humane World Privacy Policy

☐ I have read and accept the terms of the privacy statement

## General Details

*** Your primary affiliation category**

- Academia
- Contract research organization
- Governmental/public institution
- Clinic or hospital
- Industry
- Non Governmental Organisation
- Other

* Please, specify

*** What is your primary role?**

- Clinician
- Data scientist
- Group leader/coordinator
- Other
- PhD student
- Post-doc researcher
- Regulatory decision-maker
- Senior scientist/investigator

Specify your role

*** What is the primary country where you work?**

- AF - Afghanistan
- AL - Albania
- DZ - Algeria
- AD - Andorra
- AO - Angola
- AG - Antigua and Barbuda
- AR - Argentina
- AM - Armenia
- AU - Australia
- AT - Austria
- AZ - Azerbaijan
- BS - Bahamas

- BH - Bahrain
- BD - Bangladesh
- BB - Barbados
- BY - Belarus
- BE - Belgium
- BZ - Belize
- BJ - Benin
- BT - Bhutan
- BO - Bolivia
- BA - Bosnia and Herzegovina
- BW - Botswana
- BR - Brazil
- BN - Brunei Darussalam
- BG - Bulgaria
- BF - Burkina Faso
- BI - Burundi
- CV - Cabo Verde
- KH - Cambodia
- CM - Cameroon
- CA - Canada
- CF - Central African Republic
- TD - Chad
- CL - Chile
- CN - China
- CO - Colombia
- KM - Comoros
- CG - Congo
- CR - Costa Rica
- CI - Côte D'Ivoire
- HR - Croatia
- CU - Cuba
- CY - Cyprus
- CZ - Czechia
- CD - Democratic Republic of the Congo
- DK - Denmark
- DJ - Djibouti
- DM - Dominica
- DO - Dominican Republic
- EC - Ecuador
- EG - Egypt
- SV - El Salvador
- GQ - Equatorial Guinea

- ER - Eritrea
- EE - Estonia
- SZ - Eswatini
- ET - Ethiopia
- FJ - Fiji
- FI - Finland
- FR - France
- GA - Gabon
- GM - Gambia
- GE - Georgia
- DE - Germany
- GH - Ghana
- GR - Greece
- GD - Grenada
- GT - Guatemala
- GN - Guinea
- GW - Guinea Bissau
- GY - Guyana
- HT - Haiti
- HN - Honduras
- HU - Hungary
- IS - Iceland
- IN - India
- ID - Indonesia
- IR - Iran
- IQ - Iraq
- IE - Ireland
- IL - Israel
- IT - Italy
- JM - Jamaica
- JP - Japan
- JO - Jordan
- KZ - Kazakhstan
- KE - Kenya
- KI - Kiribati
- KW - Kuwait
- KG - Kyrgyzstan
- LA - Laos
- LV - Latvia
- LB - Lebanon
- LS - Lesotho
- LR - Liberia

- LY - Libya
- LI - Liechtenstein
- LT - Lithuania
- LU - Luxembourg
- MG - Madagascar
- MW - Malawi
- MY - Malaysia
- MV - Maldives
- ML - Mali
- MT - Malta
- MH - Marshall Islands
- MR - Mauritania
- MU - Mauritius
- MX - Mexico
- FM - Micronesia
- MC - Monaco
- MN - Mongolia
- ME - Montenegro
- MA - Morocco
- MZ - Mozambique
- MM - Myanmar
- NA - Namibia
- NR - Nauru
- NP - Nepal
- NL - Netherlands
- NZ - New Zealand
- NI - Nicaragua
- NE - Niger
- NG - Nigeria
- KP - North Korea
- MK - North Macedonia
- NO - Norway
- OM - Oman
- PK - Pakistan
- PW - Palau
- PA - Panama
- PG - Papua New Guinea
- PY - Paraguay
- PE - Peru
- PH - Philippines
- PL - Poland
- PT - Portugal

- QA - Qatar
- MD - Republic of Moldova
- RO - Romania
- RU - Russian Federation
- RW - Rwanda
- KN - Saint Kitts and Nevis
- LC - Saint Lucia
- VC - Saint Vincent and the Grenadines
- WS - Samoa
- SM - San Marino
- ST - Sao Tome and Principe
- SA - Saudi Arabia
- SN - Senegal
- RS - Serbia
- SC - Seychelles
- SL - Sierra Leone
- SG - Singapore
- SK - Slovakia
- SI - Slovenia
- SB - Solomon Islands
- SO - Somalia
- ZA - South Africa
- KR - South Korea
- SS - South Sudan
- ES - Spain
- LK - Sri Lanka
- SD - Sudan
- SR - Suriname
- SE - Sweden
- CH - Switzerland
- SY - Syrian Arab Republic
- TJ - Tajikistan
- TZ - Tanzania
- TH - Thailand
- TL - Timor-Leste
- TG - Togo
- TO - Tonga
- TT - Trinidad and Tobago
- TN - Tunisia
- TR - Türkiye
- TM - Turkmenistan
- TV - Tuvalu

- UG - Uganda
- UA - Ukraine
- AE - United Arab Emirates
- GB - United Kingdom
- US - United States of America
- UY - Uruguay
- UZ - Uzbekistan
- VU - Vanuatu
- VE - Venezuela
- VN - Viet Nam
- YE - Yemen
- ZM - Zambia
- ZW - Zimbabwe

## Survey

***Q1: How important do you think is having tool(s) to assess translational relevance of animals models?**

- Highly important
- Important
- Somewhat important
- Not important
- Not sure

***Q2: Were you already aware of the aforementioned tools/approaches to assess translational relevance of animals models?**

*I knew or read about:*

- RAA (clinical Relevance Assessment of Animal preclinical research)
- PATH (Preclinical Assessment for Translation to Humans)
- FIMD (Framework to Identify optimal animal Models for efficacy assessment in Drug development)
- AMQA (Animal Model Quality Assessment framework)
- AMF (Animal Model Framework)
- GRADE (Grading of Recommendations, Assessment, Development, and Evaluation)
- SYRCLE's risk of bias tool
- Translatability Score
- The 'Replacement Checklist'
- No, I didn't know these tools or frameworks before
- Other tools or approaches

*If you knew about other tools or approaches, can you list them here and provide any relevant link?

*500 character(s) maximum*

**Q3: In your opinion, how important are the following criteria when evaluating the translational value of animal models in preclinical research?**

| | 1 Not important | 2 Somewhat important | 3 Important | 4 Very important | Not sure |
|---|---|---|---|---|---|
| * Relevance of the model to mimic human pathophysiology | ○ | ○ | ○ | ○ | ○ |
| * Predictive validity for human clinical outcomes | ○ | ○ | ○ | ○ | ○ |
| * Mechanistic similarity to human biology | ○ | ○ | ○ | ○ | ○ |
| * Historical success rate in predicting human responses | ○ | ○ | ○ | ○ | ○ |
| * Species-specific differences in pharmacokinetics/pharmacodynamics (PK /PD) | ○ | ○ | ○ | ○ | ○ |
| * Availability and quality of comparative human data | ○ | ○ | ○ | ○ | ○ |
| * Reproducibility and robustness of results in the model | ○ | ○ | ○ | ○ | ○ |
| * Ethical considerations and severity of procedures | ○ | ○ | ○ | ○ | ○ |
| * Model standardization and characterization level | ○ | ○ | ○ | ○ | ○ |

| | | | | | |
|---|---|---|---|---|---|
| * Alignment with regulatory expectations or guidance | ◯ | ◯ | ◯ | ◯ | ◯ |
| * Generalizability of the model to diverse human populations (e.g., sex, age, genetics) | ◯ | ◯ | ◯ | ◯ | ◯ |
| * Ability to measure clinically relevant endpoints | ◯ | ◯ | ◯ | ◯ | ◯ |
| * Duration and stage of disease modeled | ◯ | ◯ | ◯ | ◯ | ◯ |
| * Cost-effectiveness relative to translational gain | ◯ | ◯ | ◯ | ◯ | ◯ |
| Other | ◯ | ◯ | ◯ | ◯ | ◯ |

* Please, specify

*500 character(s) maximum*

* **Q4: In your opinion, what are the potential limitations associated with tools that assess the translational value of animal models?**

*between 1 and 7 choices*

- [ ] Lack of consensus on which criteria to include
- [ ] Limited availability of high-quality comparative human data
- [ ] Insufficient transparency in scoring or weighting methods
- [ ] Over-reliance on subjective expert opinion
- [ ] Limited applicability across different disease areas
- [ ] Poor standardization or reproducibility of assessment results
- [ ] Lack of integration with regulatory or policy frameworks
- [ ] Limited usability or accessibility for non-experts
- [ ] Failure to capture ethical considerations adequately
- [ ] Tools may be biased toward preserving the status quo (animal use)
- [ ] Difficulties in validating the accuracy of the tool's outcomes
- [ ] Inadequate updates or maintenance as science evolves
- [ ] Complexity of implementation in real-world decision-making (e.g. by research institute ethical committee or funding bodies)
- [ ] Insufficient stakeholder involvement in tool development
- [ ] Limited transparency about funding sources or potential conflicts of interest
- [ ] Not sure
- [ ] Other

* Please, specify

* **Q5: In what ways could tools that assess the translational value of animal models be beneficial to research institutions and/or funding bodies?**

*between 1 and 7 choices*

- [ ] Improve decision-making in selecting appropriate models for specific research goals
- [ ] Increase transparency and accountability in research design and funding allocation
- [ ] Encourage critical evaluation of animal model limitations
- [ ] Support decision making before initiating clinical trials
- [ ] Support alignment with regulatory science and innovation policies
- [ ] Facilitate transition toward human-centric or non-animal methods
- [ ] Optimize resource use by reducing investment in low-translational models
- [ ] Strengthen the scientific justification in grant applications or ethics reviews
- [ ] Promote reproducibility and robustness in preclinical research

- [ ] Enable better tracking of research outcomes and translational success
- [ ] Contribute to institutional reputational gains by promoting responsible science
- [ ] Inform strategic planning and research portfolio management
- [ ] Foster interdisciplinary collaboration between biologists, clinicians, and data scientists
- [ ] Enhance compliance with 3Rs principles
- [ ] Support policy advocacy for evidence-based research funding reforms
- [ ] Other

* Please, specify

*200 character(s) maximum*

*** Q6: Do you believe the scoring criteria adopted in tools to evaluate the translatability of preclinical animal models could also be applicable to assess the translatability of non-animal models (NAMs)?**

- ◯ Yes, fully applicable without modifications
- ◯ Yes, but only partially — some criteria would need to be adapted
- ◯ No, NAMs require entirely different assessment criteria
- ◯ Not sure / I don’t have enough information

(not mandatory) Please explain your answer or suggest which criteria might differ

*1000 character(s) maximum*

**Q8: To what extent do you agree that the following issues represent major sources of bias in preclinical studies?**

| | 1 Disagree | 2 Somewhat agree | 3 Agree | 4 Completely agree | Not sure |
|---|---|---|---|---|---|
| * Lack of randomization of animals into experimental groups | ◯ | ◯ | ◯ | ◯ | ◯ |
| * Lack of blinding of investigators during outcome assessment | ◯ | ◯ | ◯ | ◯ | ◯ |
| * Selective outcome reporting | ◯ | ◯ | ◯ | ◯ | ◯ |
| * Publication bias (e.g., only positive studies are published) | ◯ | ◯ | ◯ | ◯ | ◯ |

| | | | | | |
|---|---|---|---|---|---|
| * Small sample sizes leading to underpowered studies | | | | | |
| * Poor or inconsistent study design | | | | | |
| * Lack of preregistration or protocol transparency | | | | | |
| * Inadequate statistical analysis or p-hacking | | | | | |
| * Conflicts of interest influencing study outcomes | | | | | |
| * Incomplete reporting of negative or null results | | | | | |
| * Overgeneralization of species-specific effects to humans | | | | | |
| * Poor reproducibility or failure to replicate findings | | | | | |
| * Overinterpretation or overselling of results to suggest human relevance | | | | | |
| * Lack of independent validation of results | | | | | |
| * Inadequate peer review or oversight during study design | | | | | |
| Other | | | | | |

* Please, specify

**Q9: To what extent should each of the following aspects be scored to predict the translational success of a preclinical model (animal or non-animal)?**

| | 1 Not important | 2 Somewhat important | 3 Important | 4 Very important | Not sure |
|---|---|---|---|---|---|
| * Biological relevance to human pathophysiology | | | | | |
| * Predictive validity (e.g., efficacy, toxicity outcomes in humans) | | | | | |
| * Face validity (visual/phenotypic similarity to human disease) | | | | | |

| | | | | | |
|---|---|---|---|---|---|
| * Construct validity (alignment of model's design with human disease biology) | ◯ | ◯ | ◯ | ◯ | ◯ |
| * Clinical concordance (historical alignment with clinical data) | ◯ | ◯ | ◯ | ◯ | ◯ |
| * Reproducibility across labs or studies | ◯ | ◯ | ◯ | ◯ | ◯ |
| * Robustness of statistical methods and experimental design | ◯ | ◯ | ◯ | ◯ | ◯ |
| * Standardization and transparency of protocols | ◯ | ◯ | ◯ | ◯ | ◯ |
| * Transparency of reporting and data availability | ◯ | ◯ | ◯ | ◯ | ◯ |
| * Absence of known bias or conflicts of interest | ◯ | ◯ | ◯ | ◯ | ◯ |
| * Inclusion of human-relevant biomarkers or endpoints | ◯ | ◯ | ◯ | ◯ | ◯ |
| * Integration with human data (e.g., omics, clinical data) | ◯ | ◯ | ◯ | ◯ | ◯ |
| * External validation or benchmarking against clinical data | ◯ | ◯ | ◯ | ◯ | ◯ |
| * Level of mechanistic insight provided | ◯ | ◯ | ◯ | ◯ | ◯ |
| * Consideration of biological variability (sex, age, genetics, comorbidities) | ◯ | ◯ | ◯ | ◯ | ◯ |
| * Ethical acceptability and alignment with the 3Rs | ◯ | ◯ | ◯ | ◯ | ◯ |
| * Technical readiness level or scalability | ◯ | ◯ | ◯ | ◯ | ◯ |
| * Level of regulatory recognition or acceptance | ◯ | ◯ | ◯ | ◯ | ◯ |
| Other | ◯ | ◯ | ◯ | ◯ | ◯ |

* Please, specify

*200 character(s) maximum*

**Q10: Please rate the importance of each criterion for inclusion in an ideal checklist to evaluate non-animal models (NAMs) as replacements for animal testing, considering the need for a fair comparison with animal models.**

| | 1 Not important | 2 Somewhat important | 3 Important | 4 Very important | Not sure |
|---|---|---|---|---|---|
| * Biological and mechanistic relevance to human disease compared to animal models | ◯ | ◯ | ◯ | ◯ | ◯ |
| * Predictive accuracy for human outcomes relative to animal model performance | ◯ | ◯ | ◯ | ◯ | ◯ |
| * Reproducibility and robustness across laboratories versus variability in animal studies | ◯ | ◯ | ◯ | ◯ | ◯ |
| * Transparency and completeness of protocols and reporting for NAMs compared to animal studies | ◯ | ◯ | ◯ | ◯ | ◯ |
| * Ethical considerations and alignment with 3Rs principles beyond animal use | ◯ | ◯ | ◯ | ◯ | ◯ |
| * Incorporation of human-relevant endpoints and biomarkers that animal models may lack | ◯ | ◯ | ◯ | ◯ | ◯ |
| * Scalability and practical feasibility for routine use in comparison to animal testing | ◯ | ◯ | ◯ | ◯ | ◯ |
| * Independent validation and benchmarking of NAMs against both animal data and clinical outcomes | ◯ | ◯ | ◯ | ◯ | ◯ |
| * Regulatory acceptance and compatibility relative to existing animal testing frameworks | ◯ | ◯ | ◯ | ◯ | ◯ |
| * Ability to model biological variability (e.g., sex, age, genetics) similarly or better than animals | ◯ | ◯ | ◯ | ◯ | ◯ |
| * Cost-effectiveness and resource requirements compared to animal experiments | ◯ | ◯ | ◯ | ◯ | ◯ |
| * Availability of user training, technical support, and infrastructure compared to animal facilities | ◯ | ◯ | ◯ | ◯ | ◯ |
| Other | ◯ | ◯ | ◯ | ◯ | ◯ |

* Are there any additional criteria you believe should be included in such a checklist to fairly compare NAMs with animal models? Please, specify

*500 character(s) maximum*

* As a follow-up of your contribution to this survey, are you willing to be contacted again for future activities?

- Yes (please provide your contact details below or send an email to fpistollato@humaneworld.org)
- No

First and Last name

Email

**Thank you for your participation!**
