# Supplementary material for: Awareness and perspectives of translational assessment tools in preclinical research: insights from a survey
Source: NAM J. 2026 Oct 13;2:100146. doi: 10.1016/j.namjnl.2026.100146 (PMC13696333; doi:10.1016/j.namjnl.2026.100146)
Supplement: Supplementary file 2 [file mmc2.docx]

Supplementary File 2

**Awareness and perspectives of translational assessment tools in preclinical research: insights from a survey**

Francesca Pistollato, Ignacio J. Tripodi, Fabia Furtmann, Marco Straccia, and Helder Constantino

*** Correspondence:** Francesca Pistollato: [fpistollato@humaneworld.org](mailto:fpistollato@humaneworld.org)

**Completed CROSS and CHERRIES Reporting Checklists**

**Table S2.1. Checklist for Reporting Of Survey Studies (CROSS)**

CROSS is a reporting guideline for web- and non-web-based survey studies. Where an item was not applicable or information was unavailable, this is stated explicitly.

| **Section/topic** | **Item** | **CROSS item description** | **Reported in / study-specific note** |
| --- | --- | --- | --- |
| Title and abstract | 1a | State the word “survey” along with a commonly used term in title or abstract to introduce the study’s design. | Title |
| Title and abstract | 1b | Provide an informative summary in the abstract, covering background, objectives, methods, findings/results, interpretation/discussion, and conclusions. | Abstract |
| Background | 2 | Provide a background about the rationale of study, what has been previously done, and why this survey is needed. | Introduction |
| Purpose/aim | 3 | Identify specific purposes, aims, goals, or objectives of the study. | Introduction – study aims/objectives |
| Study design | 4 | Specify the study design in the Methods section with a commonly used term (e.g., cross-sectional or longitudinal). | Methods – study design |
| Data collection methods | 5a | Describe the questionnaire (e.g., number of sections, number of questions, number and names of instruments used). | Methods – questionnaire development and administration; survey questionnaire |
| Data collection methods | 5b | Describe all questionnaire instruments that were used in the survey to measure particular concepts. Report target population, reported validity and reliability information, scoring/classification procedure, and reference links (if any). | Methods – questionnaire development and testing. The questionnaire was study-specific, exploratory, and was not adapted from an existing or validated instrument; no formal psychometric scale was used. |
| Data collection methods | 5c | Provide information on pretesting of the questionnaire, if performed (in the article or in an online supplement). Report the method of pretesting, number of times questionnaire was pre-tested, number and demographics of participants used for pretesting, and the level of similarity of demographics between pre-testing participants and sample population. | Methods – questionnaire development and testing. Draft questionnaire reviewed in two rounds by researchers/subject-matter experts and pilot-tested by five researchers for clarity, relevance and comprehensibility; revised based on feedback. |
| Data collection methods | 5d | Questionnaire, if possible, should be fully provided (in the article, or as appendices or as an online supplement). | Survey questionnaire / supplementary materials |
| Sample characteristics | 6a | Describe the study population (i.e., background, locations, eligibility criteria for participant inclusion in survey, exclusion criteria). | Methods – survey dissemination/study population. Targeted researchers and other professionals active in preclinical, nonclinical, translational and related biomedical research; no additional individual-level eligibility screening was applied. |
| Sample characteristics | 6b | Describe the sampling techniques used (e.g., single stage or multistage sampling, simple random sampling, stratified sampling, cluster sampling, convenience sampling). Specify the locations of sample participants whenever clustered sampling was applied. | Methods – survey dissemination/study population; Supplementary File 2. Targeted, non-probability recruitment using PubMed-derived corresponding-author email lists, LinkedIn dissemination and secondary sharing. |
| Sample characteristics | 6c | Provide information on sample size, along with details of sample size calculation. | Methods – study population/sample. No a priori sample-size calculation was performed because the survey was exploratory and used targeted, non-probability recruitment. |
| Sample characteristics | 6d | Describe how representative the sample is of the study population (or target population if possible), particularly for population-based surveys. | Discussion – limitations/generalizability. The non-probability, link-shareable recruitment strategy does not permit the sample to be considered representative of the wider preclinical research community. |
| Survey administration | 7a | Provide information on modes of questionnaire administration, including the type and number of contacts, the location where the survey was conducted (e.g., outpatient room or by use of online tools, such as SurveyMonkey). | Methods – survey administration/dissemination; Supplementary File 2. Web survey hosted on EUSurvey; launched via LinkedIn and disseminated through four PubMed-derived email rounds. |
| Survey administration | 7b | Provide information of survey’s time frame, such as periods of recruitment, exposure, and follow-up days. | Methods – survey administration; Supplementary File 2. Survey open 17 June 2025-20 January 2026; email dissemination rounds on 9 September, 29 September, 14 October and 18 November 2025. |
| Survey administration | 7c | Provide information on the entry process: for non-web-based surveys, provide approaches to minimize human error in data entry; for web-based surveys, provide approaches to prevent “multiple participation” of participants. | Methods – survey completion/data quality. EUSurvey mechanisms were used to restrict/manage multiple submissions; no IP-based duplicate check or log-file analysis was performed. Duplicate email addresses were removed from mailing lists before dissemination. |
| Study preparation | 8 | Describe any preparation process before conducting the survey (e.g., interviewers’ training process, advertising the survey). | Methods – questionnaire development/testing and survey dissemination. Two rounds of expert review and pilot testing preceded launch; the survey was advertised via LinkedIn and targeted email dissemination. |
| Ethical considerations | 9a | Provide information on ethical approval for the survey if obtained, including informed consent, institutional review board [IRB] approval, Helsinki declaration, and good clinical practice [GCP] declaration (as appropriate). | Methods – ethical considerations. No prospective REC/IRB approval or formal exemption determination was sought; the survey was considered minimal-risk and non-interventional. Participant information, privacy acknowledgement and consent process are described. |
| Ethical considerations | 9b | Provide information about survey anonymity and confidentiality and describe what mechanisms were used to protect unauthorized access. | Methods – ethical considerations/data management. Identifying information was optional; exported data were stored on a secure HWA-controlled drive with access restricted to two study authors. Three-year retention followed by secure deletion is specified. |
| Statistical analysis | 10a | Describe statistical methods and analytical approach. Report the statistical software that was used for data analysis. | Methods – statistical analysis |
| Statistical analysis | 10b | Report any modification of variables used in the analysis, along with reference (if available). | Methods – statistical analysis, as applicable |
| Statistical analysis | 10c | Report details about how missing data was handled. Include rate of missing items, missing data mechanism (i.e., MCAR, MAR, or MNAR), and methods used to deal with missing data (e.g., multiple imputation). | Methods – survey completion/data quality. Mandatory items were required for submission; partial/unsubmitted questionnaires were not retained. No missing data occurred for mandatory items and no imputation was performed. |
| Statistical analysis | 10d | State how non-response error was addressed. | Methods – Data analysis and presentation. Conventional non-response/completion metrics could not be fully assessed because partial/unsubmitted questionnaires and unique visitor counts were unavailable and the survey link could be shared. |
| Statistical analysis | 10e | For longitudinal surveys, state how loss to follow-up was addressed. | Not applicable – cross-sectional survey |
| Statistical analysis | 10f | Indicate whether any methods such as weighting of items or propensity scores have been used to adjust for non-representativeness of the sample. | Methods – statistical analysis. No weighting, propensity-score adjustment or other correction for non-representativeness was performed. |
| Statistical analysis | 10g | Describe any sensitivity analysis conducted. | Not applicable / none conducted |
| Respondent characteristics | 11a | Report numbers of individuals at each stage of the study. Consider using a flow diagram, if possible. | Methods/Results – recruitment and respondent numbers; Supplementary File 2. Mailing-list sizes and email bounce information are reported; only submitted questionnaires were retained by EUSurvey. |
| Respondent characteristics | 11b | Provide reasons for non-participation at each stage, if possible. | Not available. EUSurvey did not retain partial/unsubmitted questionnaires; reasons for non-participation were not collected. |
| Respondent characteristics | 11c | Report response rate, present the definition of response rate or the formula used to calculate response rate. | Methods and limitations. A conventional response rate could not be reliably calculated because of email bounces, secondary sharing of the survey link, and the absence of unique visitor/partial-response data. |
| Respondent characteristics | 11d | Provide information to define how unique visitors are determined. Report number of unique visitors along with relevant proportions (e.g., view proportion, participation proportion, completion proportion). | Not available. Unique visitor counts were not retained/available; view, participation and completion proportions could therefore not be calculated. |
| Descriptive results | 12 | Provide characteristics of study participants, as well as information on potential confounders and assessed outcomes. | Results – respondent characteristics and survey findings |
| Main findings | 13a | Give unadjusted estimates and, if applicable, confounder-adjusted estimates along with 95% confidence intervals and p values. | Method / Results – survey findings / Limitations. Descriptive results are reported as frequencies and percentages, as appropriate. No inferential statistical analyses, hypothesis testing, or adjusted estimates were performed, given the exploratory and descriptive nature of the study and the limited sample size. |
| Main findings | 13b | For multivariable analysis, provide information on the model building process, model fit statistics, and model assumptions (as appropriate). | Not applicable – no multivariable analyses were performed. |
| Main findings | 13c | Provide details about any sensitivity analysis performed. If there are considerable amount of missing data, report sensitivity analyses comparing the results of complete cases with that of the imputed dataset (if possible). | Not applicable – no sensitivity analyses or missing-data imputation were performed. |
| Limitations | 14 | Discuss the limitations of the study, considering sources of potential biases and imprecisions, such as non-representativeness of sample, study design, important uncontrolled confounders. | Discussion – limitations. Potential limitations related to geographical imbalance, non-probability sampling, self-selection/non-response bias, limited sample size, and generalisability are discussed in the Limitations section. |
| Interpretations | 15 | Give a cautious overall interpretation of results, based on potential biases and imprecisions and suggest areas for future research. | Discussion – limitations and conclusions |
| Generalizability | 16 | Discuss the external validity of the results. | Discussion – limitations/generalizability |
| Role of the funding source | 17 | State whether any funding organization has had any roles in the survey’s design, implementation, and analysis. | Funding statement / declarations |
| Conflict of interest | 18 | Declare any potential conflict of interest. | Conflict of interest / declarations |
| Acknowledgements | 19 | Provide names of organizations/persons that are acknowledged along with their contribution to the research. | Acknowledgements, as applicable |

**Table S2.2. Checklist for Reporting Results of Internet E-Surveys (CHERRIES)**

| **CHERRIES category** | **Checklist item** | **Study-specific reporting** | **Location in manuscript/materials** |
| --- | --- | --- | --- |
| Design | Describe survey design | Target population: researchers and other professionals active in preclinical, nonclinical, translational and related biomedical research. A targeted, non-probability recruitment strategy was used. Potential participants were primarily identified through corresponding-author email addresses retrieved from PubMed using predefined searches; the invitation could also be shared with other relevant professionals. | Methods – Survey dissemination/study population; Supplementary File 2 |
| IRB approval and informed consent | IRB approval | No prospective research ethics committee/IRB approval or formal exemption determination was sought. The survey received no U.S. federal funding or support. The study was considered a minimal-risk, non-interventional professional survey. | Methods – Ethical considerations |
| IRB approval and informed consent | Informed consent | Before proceeding, participants were informed of the organisation conducting the survey, its purpose and scope, the expected completion time (approximately 15 minutes), and that responses would remain anonymous. Participants were provided with the Humane World for Animals Privacy Policy and were required to confirm that they had read and accepted it. No separate written research-consent form was used; completion of the survey after reviewing this information was considered to indicate implied consent to participate. | Methods – Ethical considerations; participant-facing survey introduction |
| IRB approval and informed consent | Data protection | Identifying information was not required for participation. Respondents could optionally provide their name and email address at the end of the survey if they wished to be contacted regarding future activities; these details were technically linked to the corresponding survey responses. Exported data were stored on a secure Humane World for Animals-controlled drive, with access restricted to two study authors (FP and FF). Data will be retained for three years and subsequently securely deleted. EUSurvey and Humane World for Animals privacy safeguards applied. | Methods – Ethical considerations/data management |
| Development and pre-testing | Development and testing | The questionnaire was developed by the authors specifically for the objectives of this study and was not adapted from an existing or validated instrument. During its development, the authors reviewed previous surveys addressing researchers’ perspectives on experimental models and related aspects of biomedical research, which provided general methodological and contextual background but differed substantially in their specific objectives and content. The questionnaire was not developed around a formal theoretical or psychometric framework; rather, its domains and individual questions were defined according to the exploratory objectives of the study and the existing landscape of tools and frameworks for assessing translational value. The draft questionnaire underwent two rounds of review involving researchers and subject-matter experts, including experts who had participated in a previous BioMed21 workshop on approaches for evaluating the translational value of animal models in preclinical research. It was subsequently pilot-tested by five researchers to assess the clarity, relevance and comprehensibility of the questions and response options and was revised based on their feedback. The questionnaire was developed and administered in English. | Methods – Questionnaire development and testing |
| Recruitment | Open survey versus closed survey | The survey link was distributed primarily by email to a targeted list of potential participants identified through PubMed. Recipients were also invited to share the link with representatives of preclinical research organisations who might be interested. The survey was therefore open/link-shareable rather than a strictly closed or password-protected survey. | Methods – Survey dissemination |
| Recruitment | Contact mode | Initial contact was made electronically by email. Potential participants were identified primarily from corresponding-author email addresses retrieved from PubMed. | Methods – Survey dissemination; Supplementary File 2 |
| Recruitment | Advertising the survey | The survey was launched via a LinkedIn post and was subsequently disseminated by email using PubMed-derived mailing lists. Search strings, dissemination dates and numbers of email addresses are documented in Supplementary File 2. The survey introduction also allowed recipients to share the survey link with other relevant professionals. | Methods – Survey dissemination; Supplementary File 2 |
| Survey administration | Web/E-mail | The web-based questionnaire was hosted on the European Commission EUSurvey platform. Invitations were disseminated electronically, and responses were captured by EUSurvey and subsequently exported for analysis. | Methods – Survey administration |
| Survey administration | Context | The questionnaire was hosted on EUSurvey. Recruitment primarily targeted researchers and professionals identified through literature-based searches rather than visitors to a topical website. Because recipients could forward the link, some respondents may have accessed the survey through secondary sharing. | Methods – Survey dissemination |
| Survey administration | Mandatory/voluntary | Participation was voluntary. Designated mandatory items had to be answered before final submission. | Methods – Ethical considerations; survey completion |
| Survey administration | Incentives | No incentives were offered for participation. | Methods – Survey administration |
| Survey administration | Time/Date | The survey was launched on 17 June 2025 via a LinkedIn post and closed on 20 January 2026. Email dissemination rounds were conducted on 9 September, 29 September, 14 October and 18 November 2025. | Methods – Survey administration; Supplementary File 2 |
| Survey administration | Randomization of items or questionnaires | Questions were presented in a fixed order. | Methods – Questionnaire administration |
| Survey administration | Adaptive questioning | Adaptive questioning was not used. | Methods – Questionnaire administration |
| Survey administration | Number of items | The questionnaire contained predefined-response and open-text items, all displayed on one page. | Methods / survey questionnaire |
| Survey administration | Number of screens (pages) | One page. | Methods / survey questionnaire |
| Survey administration | Completeness check | EUSurvey was configured so that all mandatory questions had to be answered before a questionnaire could be submitted. Partial or unsubmitted questionnaires were not retained by the platform and were not downloadable. Submitted questionnaires therefore had no missing data for mandatory items, and no imputation was required. | Methods – Survey completion/data quality |
| Survey administration | Review step | Respondents could navigate back, review and change their answers before final submission. | Methods – Questionnaire administration |
| Response metrics | Unique site visitor | Unique survey visitor counts were not available from the information retained for analysis. | Methods/Results – Response metrics |
| Response metrics | View rate | Not calculable because unique visitors to the survey/hosting site were not retained or available. | Results / CHERRIES checklist |
| Response metrics | Participation rate | Not calculable because the number of unique visitors to the first survey/consent page who did not proceed was not retained or available. | Results / CHERRIES checklist |
| Response metrics | Completion rate | Not calculable because EUSurvey did not retain partial or unsubmitted questionnaires; therefore, the number of individuals initiating but not completing the survey was unknown. | Results / CHERRIES checklist |
| Preventing multiple entries | Cookies used | EUSurvey provides mechanisms to restrict or manage multiple submissions. | Methods – Survey completion/data quality |
| Preventing multiple entries | IP check | No IP-based duplicate check was performed. | Methods / CHERRIES checklist |
| Preventing multiple entries | Log file analysis | No log-file analysis for duplicate responses was performed. | Methods / CHERRIES checklist |
| Preventing multiple entries | Registration | No respondent registration or login was required. Survey invitations used a link that recipients could share; registration-based duplicate prevention was therefore not used. | Methods / CHERRIES checklist |
| Analysis | Handling of incomplete questionnaires | Only submitted questionnaires were available for analysis. Partial or unsubmitted questionnaires were not retained by EUSurvey and were therefore unavailable for inclusion. Mandatory questions had to be completed for submission. | Methods – Survey completion/data quality |
| Analysis | Questionnaires submitted with an atypical timestamp | No responses were excluded on the basis of completion time. | Methods – Data analysis |
| Analysis | Statistical correction | No weighting, propensity-score adjustment or other correction for the non-probability/non-representative sample was performed. | Methods – Statistical analysis; Discussion – Limitations |

**Checklist sources:** Sharma A, et al. A Consensus-Based Checklist for Reporting of Survey Studies (CROSS). J Gen Intern Med. 2021;36(10):3179–3187. doi:10.1007/s11606-021-06737-1. Eysenbach G. Improving the Quality of Web Surveys: The Checklist for Reporting Results of Internet E-Surveys (CHERRIES). J Med Internet Res. 2004;6(3):e34. doi:10.2196/jmir.6.3.e34.

**Table S2.3. Perceived limitations of translational assessment tools by primary affiliation.** Values represent the number and percentage of respondents within each affiliation category selecting each limitation. Multiple responses were permitted; therefore, percentages do not sum to 100%. Findings should be interpreted descriptively given the small and uneven subgroup sizes.

| Q4 (limitations of translational assessment tools) | Overall (n=105) | Academia (n=58) | Government/public (n=15) | Clinic/hospital (n=10) | NGO (n=8) | CRO (n=8) | Industry (n=5) | Other affiliation (n=1) |
| --- | --- | --- | --- | --- | --- | --- | --- | --- |
| Poor standardization or reproducibility of assessment results | 53 (50.5%) | 28 (48.3%) | 9 (60.0%) | 3 (30.0%) | 4 (50.0%) | 4 (50.0%) | 4 (80.0%) | 1 (100.0%) |
| Limited availability of high-quality comparative human data | 52 (49.5%) | 30 (51.7%) | 8 (53.3%) | 5 (50.0%) | 3 (37.5%) | 2 (25.0%) | 3 (60.0%) | 1 (100.0%) |
| Lack of consensus on which criteria to include | 52 (49.5%) | 25 (43.1%) | 10 (66.7%) | 6 (60.0%) | 3 (37.5%) | 5 (62.5%) | 3 (60.0%) | 0 (0.0%) |
| Complexity of implementation in real-world decision-making (e.g. by research institute ethical committee or funding bodies) | 41 (39.0%) | 20 (34.5%) | 6 (40.0%) | 4 (40.0%) | 5 (62.5%) | 2 (25.0%) | 3 (60.0%) | 1 (100.0%) |
| Difficulties in validating the accuracy of the tool's outcomes | 41 (39.0%) | 24 (41.4%) | 6 (40.0%) | 3 (30.0%) | 3 (37.5%) | 3 (37.5%) | 1 (20.0%) | 1 (100.0%) |
| Over-reliance on subjective expert opinion | 41 (39.0%) | 26 (44.8%) | 5 (33.3%) | 3 (30.0%) | 4 (50.0%) | 1 (12.5%) | 2 (40.0%) | 0 (0.0%) |
| Insufficient transparency in scoring or weighting methods | 31 (29.5%) | 14 (24.1%) | 5 (33.3%) | 5 (50.0%) | 2 (25.0%) | 2 (25.0%) | 2 (40.0%) | 1 (100.0%) |
| Lack of integration with regulatory or policy frameworks | 30 (28.6%) | 11 (19.0%) | 7 (46.7%) | 4 (40.0%) | 2 (25.0%) | 3 (37.5%) | 3 (60.0%) | 0 (0.0%) |
| Tools may be biased toward preserving the status quo (animal use) | 25 (23.8%) | 10 (17.2%) | 5 (33.3%) | 1 (10.0%) | 6 (75.0%) | 2 (25.0%) | 0 (0.0%) | 1 (100.0%) |
| Insufficient stakeholder involvement in tool development | 24 (22.9%) | 15 (25.9%) | 3 (20.0%) | 1 (10.0%) | 0 (0.0%) | 4 (50.0%) | 1 (20.0%) | 0 (0.0%) |
| Failure to capture ethical considerations adequately | 23 (21.9%) | 10 (17.2%) | 3 (20.0%) | 1 (10.0%) | 5 (62.5%) | 2 (25.0%) | 1 (20.0%) | 1 (100.0%) |
| Limited applicability across different disease areas | 20 (19.0%) | 12 (20.7%) | 3 (20.0%) | 4 (40.0%) | 0 (0.0%) | 0 (0.0%) | 1 (20.0%) | 0 (0.0%) |
| Limited usability or accessibility for non-experts | 19 (18.1%) | 7 (12.1%) | 3 (20.0%) | 3 (30.0%) | 1 (12.5%) | 3 (37.5%) | 2 (40.0%) | 0 (0.0%) |
| Limited transparency about funding sources or potential conflicts of interest | 14 (13.3%) | 10 (17.2%) | 1 (6.7%) | 0 (0.0%) | 2 (25.0%) | 1 (12.5%) | 0 (0.0%) | 0 (0.0%) |
| Inadequate updates or maintenance as science evolves | 9 (8.6%) | 2 (3.4%) | 2 (13.3%) | 1 (10.0%) | 1 (12.5%) | 2 (25.0%) | 1 (20.0%) | 0 (0.0%) |
| Not sure | 3 (2.9%) | 2 (3.4%) | 0 (0.0%) | 1 (10.0%) | 0 (0.0%) | 0 (0.0%) | 0 (0.0%) | 0 (0.0%) |
| Other | 8 (7.6%) | 5 (8.6%) | 1 (6.7%) | 0 (0.0%) | 0 (0.0%) | 1 (12.5%) | 1 (20.0%) | 0 (0.0%) |

**Table S2.4. Applicability of animal-model translational assessment criteria to NAMs, by primary professional affiliation.** Values are n (% of respondents within each affiliation category). Q6 permitted one response per respondent. Percentages may differ slightly from 100% due to rounding. Small and uneven subgroup sizes limit comparisons, therefore findings are descriptive only.

| Q6 (applicability of scoring criteria to NAMs) | Overall (N=105) | Academia (n=58) | Governmental/public institution (n=15) | Clinic or hospital (n=10) | NGO (n=8) | CRO (n=8) | Industry (n=5) | Other (n=1) |
| --- | --- | --- | --- | --- | --- | --- | --- | --- |
| Yes, fully applicable without modifications | 8 (7.6%) | 4 (6.9%) | 1 (6.7%) | 0 (0.0%) | 0 (0.0%) | 2 (25.0%) | 1 (20.0%) | 0 (0.0%) |
| Yes, but only partially — some criteria would need to be adapted | 51 (48.6%) | 23 (39.7%) | 9 (60.0%) | 6 (60.0%) | 8 (100.0%) | 2 (25.0%) | 3 (60.0%) | 0 (0.0%) |
| No, NAMs require entirely different assessment criteria | 19 (18.1%) | 12 (20.7%) | 3 (20.0%) | 0 (0.0%) | 0 (0.0%) | 3 (37.5%) | 1 (20.0%) | 0 (0.0%) |
| Not sure / I don’t have enough information | 27 (25.7%) | 19 (32.8%) | 2 (13.3%) | 4 (40.0%) | 0 (0.0%) | 1 (12.5%) | 0 (0.0%) | 1 (100.0%) |
| Total | 105 | 58 | 15 | 10 | 8 | 8 | 5 | 1 |
