# Supplementary material for: Awareness and perspectives of translational assessment tools in preclinical research: insights from a survey
Source: NAM J. 2026 Oct 13;2:100146. doi: 10.1016/j.namjnl.2026.100146 (PMC13696333; doi:10.1016/j.namjnl.2026.100146)
Supplement: Supplementary file 3 [file mmc3.docx]

Supplementary File 3

**Awareness and perspectives of translational assessment tools in preclinical research: insights from a survey**

Francesca Pistollato, Ignacio J. Tripodi, Fabia Furtmann, Marco Straccia, and Helder Constantino

*** Correspondence:** Francesca Pistollato: [fpistollato@humaneworld.org](mailto:fpistollato@humaneworld.org)

# 1 Methodological approach used to disseminate the survey via email

A simple and reproducible approach for using PubMed to generate large email lists of potential participants was used, as previously described (Khalifa M. Stud Health Technol Inform. 2019 Jul 4:262:348-351. available at <https://pubmed.ncbi.nlm.nih.gov/31349339/>).

Different sets of emails identified in Pubmed using the combinations of search terms and Boolean operators indicated in Table S1 were extracted using the email extractor (<https://www.convertcsv.com/email-extractor.htm#google_vignette>) and sent, considering as filters only English papers published in the last five years. Duplicate emails were excluded across the generated mailing lists. On average, between 10-25% of emails sent bounced back as either inexistent or not reachable.

| **Table S1:** Strings of keywords used to retrieve email addresses as described by Khalifa et al. 2019. | |
| --- | --- |
| **Keywords and Boolean operators** | **Number of emails sent (date)** |
| (preclinical research[Affiliation] OR nonclinical research[Affiliation] OR clinical[Affiliation] OR translational research[Affiliation] OR drug development[Affiliation] OR drug discovery[Affiliation] OR drug failure[Affiliation] OR drug attrition[Affiliation]) AND (translational relevance[Title/Abstract] OR non-animal methods[Title/Abstract] OR NAMs[Title/Abstract] OR animal models[Title/Abstract] OR drug failure[Title/Abstract] OR drug attrition[Title/Abstract] OR translational research[Title/Abstract] OR human-based[Title/Abstract] OR impact[Title/Abstract]) | 6,519 (Sep 9, 2025) |
| (pharma[Affiliation] OR pharmaceutical[Affiliation] OR medicine[Affiliation] OR drug[Affiliation]) AND (translational relevance[Title/Abstract] OR non-animal methods[Title/Abstract] OR NAMs[Title/Abstract] OR animal models[Title/Abstract] OR drug failure[Title/Abstract] OR drug attrition[Title/Abstract] OR translational research[Title/Abstract] OR human-based[Title/Abstract]) | 5,659 (Sep 29, 2025) |
| (disease[Affiliation] OR preclinical[Affiliation] OR clinical[Affiliation] OR drug[Affiliation] OR pharma[Affiliation]) AND (translational[Title/Abstract] OR non-animal[Title/Abstract] OR NAM[Title/Abstract] OR mice[Title/Abstract] OR rodent[Title/Abstract] OR animal[Title/Abstract] OR rat[Title/Abstract] OR drug[Title/Abstract] OR attrition[Title/Abstract] OR translation[Title/Abstract] OR human-based[Title/Abstract] OR patient[Title/Abstract] OR cohort[Title/Abstract]) | 6,119 (Oct 14, 2025) |
| (disease[Affiliation] OR preclinical[Affiliation] OR clinical[Affiliation] OR clinic[Affiliation] OR nonclinical[Affiliation] OR drug[Affiliation] OR pharma[Affiliation] OR pharmaceutical[Affiliation]) AND (translational[Title/Abstract] OR in vitro[Title/Abstract] OR in silico[Title/Abstract] OR mouse[Title/Abstract] OR rodent[Title/Abstract] OR animal[Title/Abstract] OR rat[Title/Abstract] OR monkey[Title/Abstract] OR drug[Title/Abstract] OR treatment[Title/Abstract] OR therapy[Title/Abstract] OR attrition rate[Title/Abstract] OR drug discovery[Title/Abstract]) | 4,793 (Nov 18, 2025) |

# 2 Additional comments provided by survey respondents in reply to some of the survey questions

Additional comments provided by survey respondents in reply to some of survey questions (under “others”) are reported below:

**Q2: Were you already aware of the aforementioned tools/approaches to assess translational relevance of animals models? I knew or read about:**

Others:

- BimmoH
- IAEC proposal Check list (<https://www.ncbs.res.in/sites/default/files/users/joryae/IAEC%20proposal%20Check%20list_2019_05_16.pdf>)
- REPLACE
- One centralized tool could be useful
- Papers

**Q3: In your opinion, how important are the following criteria when evaluating the translational value of animal models in preclinical research?**

Others (rate and describe the additional proposed criterion or criteria):

- Very important: Thank you for setting up this survey. Very relevant. I believe that your model can LOOK like the human disease but still be not predictive at all. What we need is 1-to-1 comparison between what works in a model and what works in humans. I also believe that many commonly used models have almost no relevance to predict human outcomes but the scientific system is so used to them and they are so accepted that everyone uses them. Scientific inertia at its finest.
- Very important: This list is biased towards a certain outcome. No model can offer generalizability and importantly no model can reasonably provide all of the above. Models need to be relevant to the specific question and need to be optimized and develop for this.
- Important: I expect the same from these programs as I expect for animal models
- Important: High quality publication acceptability
- Important: already accepted endpoint in existing regulation

**Q4: In your opinion, what are the potential limitations associated with tools that assess the translational value of animal models?**

Others:

- often systematic reviews / evidence synthesis is not a mandatory part of the process, and it should be
- Tools are presented as an replacement of an animal experiments (but they are completely different test systems. We should not aim at replacement, but develop alternative test systems to make animal experiments redundant)
- lack of incentive to evolve current traditional approaches (eg patent extension)
- Lack of expertise and funding
- Assessment tools unimportant relative to the obvious need for wider use of valid animal models
- researchers often use of improper models (models that do not represent human situation properly)
- Insufficient awareness of differences in PK between preclinical species and humans
- Lack of awareness of the importance of the context / conditions in which data are obtained, as these may influence the data (see De Lange. Mastermind Approch.. FBCNS. 2013)

**Q5: In what ways could tools that assess the translational value of animal models be beneficial to research institutions and/or funding bodies?**

Others:

- Humane: any animal studies must require extremely stringent justification
- Ensure alternative approaches (eg NAMs) are developed, validated and run in parallel to traditional in vivo approaches prior to replacing them.
- Tools unimportant relative to translational animal models
- It is a strength of basic preclinical research that it is open for novel approaches, ideas and discoveries. Pushing for pre-defined models can be disadvantageous constraint.
- Improve research protocols: measure multiple aspects systematically as a function of time and body location: have connected data- use mathematical modelling- built translational framework

**Q8: To what extent do you agree that the following issues represent major sources of bias in preclinical studies?**

Others:

- Unsure: Unclear relevance of the animal model
- Completely agree: Again this is a biased questionnaire. These questions are not specific to animal research and equally apply to all types of research.
- Completely agree: there are very few good models of psychiatric disorders, irrespective of any other consideration
- Completely agree: Lack of knowledge of animal anatomy, physiology, and pathology
- Completely agree: The quality of the animal model itself, including its ability to simulate human diseases, standardization, repeatability, and stability
- Not sure: In order to answer this question correct I need a definition for bias. Are we talking about systematic differences between groups? or also about general biases due to for example calibration errors, which may skew the result, but not the difference between groups.
- Not sure: as above- lack of connected data (systematically obtained)- all is too fragmented- not the full picture
- Agree: pharmacological proof of concept studies are not GLP studies, thus more exposed to bias/misinterpretation of the results. Generally these kind of studies are carried out in academia which has poor regulatory experience

**Q9: To what extent should each of the following aspects be scored to predict the translational success of a preclinical model (animal or non-animal)?**

Others:

- Very important: This lacks focus on fundamental research
- Not important: This is a difficult question. All the arguments are important but it is impossible to give a general answer because each tool has its own important aspects.
- Not sure: The list is comprehensive, not sure whether there are additional points to consider
- Not sure: Many of these aspects capture only part of the in vivo model analysis and this is often driven by the attractiveness of the journal a particular research scheme can be published in.

(Open question) **Are there any additional criteria you believe should be included in such a checklist to fairly compare NAMs with animal models?**

**Please, specify:**

- Anticipating and addressing "interfering effects."
- NAMs should not be compared to animal models. Animals have their values, but the goal of NAMs is to say something for the human, clinical situation/relevance. NAMs should never be compared to animals but have their own value to make the use of animal experimentation a no go.
- None yet, comprehensive questionnaire
- Again this is heavily biased towards a desired outcome. In reality all models have their own limitations and their limits. This is true for non-animal and animal modelling.
- These points betray a lack of understanding of animal models: variability in humans can be reflected in animal models, and that is not a weakness. Lack of variability will not get us anywhere, at least in psychiatry.
- I believe we should use animals better (full body context) and condense knowledge in math models to replace animals. Now a lot of money and time goes into all kinds of NAMs which should be validated ... with what? Animal work?
- The cell-type–specific limitations need to be considered, particularly whether the cells possess organ-specific functions inherent to the tissue of origin.
